# Tethered particle motion of the adaptation enzyme CheR in bacterial chemotaxis

**DOI:** 10.1016/j.isci.2023.107950

**Published:** 2023-09-17

**Authors:** Caijuan Yue, Chi Zhang, Rongjing Zhang, Junhua Yuan

**Affiliations:** 1 Hefei National Laboratory for Physical Sciences at the Microscale, and Department of Physics, University of Science and Technology of China, Hefei, Anhui 230026, China

**Keywords:** Biochemistry, Enzymology, Microbiology, Bacteriology

## Abstract

Bacteria perform chemotactic adaptation by sequential modification of multiple modifiable sites on chemoreceptors through stochastic action of tethered adaptation enzymes (CheR and CheB). To study the molecular kinetics of this process, we measured the response to different concentrations of MeAsp for the Tar-only *Escherichia coli* strain. We found a strong dependence of the methylation rate on the methylation level and established a new mechanism of adaptation kinetics due to tethered particle motion of the methylation enzyme CheR. Experiments with various lengths of the C-terminal flexible chain in the Tar receptor further validated this mechanism. The tethered particle motion resulted in a CheR concentration gradient that ensures encounter-rate matching of the sequential modifiable sites. An analytical model of multisite catalytic reaction showed that this enables robustness of methylation to fluctuations in receptor activity or cell-to-cell variations in the expression of adaptation enzymes and reduces the variation in methylation level among individual receptors.

## Highlights

• The methylation rate decreases with the methylation level for the Tar-only strain
• TPM of CheR induces the dependence of the methylation rate on methylation level
• TPM of CheR ensures encounter-rate matching of the sequential modifiable sites
• The encounter-rate matching enables robustness of methylation to noise in cells

## Graphical abstract

**Figure:**
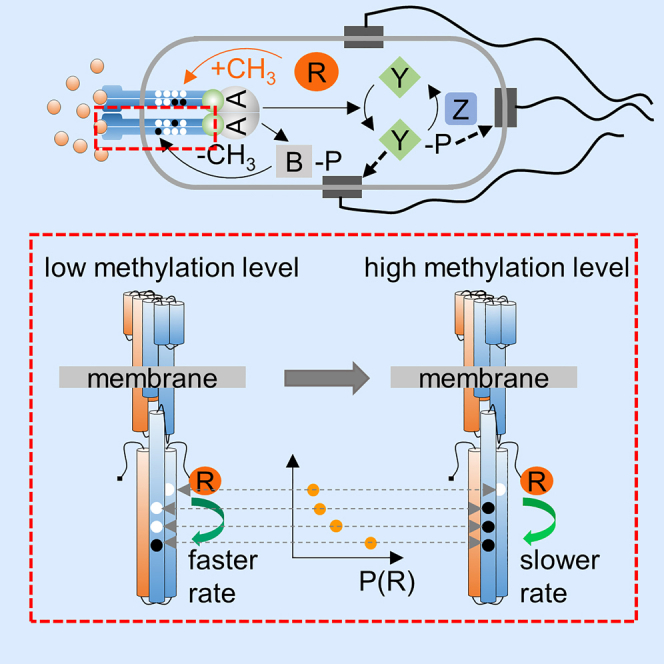

## Introduction

Intrinsically disordered regions with unstructured polypeptide chains are widely present in proteins and serve critical functions.^1^^,^^2^ They can facilitate liquid-liquid phase separation of macromolecules in cells,^3^ enable allosteric regulation for functional proteins,^4^^,^^5^ mediate forces between microtubules and neurofilaments as polymer brushes,^6^^,^^7^ or act as flexible tethers that increase the local concentration of enzymes.^8^ In the bacterial chemotaxis pathway in *Escherichia coli*, proteins with unstructured sequences are also abundant, such as the kinase CheA,^9^^,^^10^ phosphatase CheZ^11^ and the two major receptors Tar and Tsr.^12^ The function of these proteins depends strongly on the properties of the unstructured polypeptides, such as chain length and flexibility. This prompted us to systematically and quantitatively study the physiological effects of this structure on bacterial chemotaxis.

Mobile bacteria are able to track chemical gradients in their surroundings using the chemotaxis signaling transduction network.^13^^,^^14^^,^^15^ *E*. *coli*, a typical bacterium used in studies of chemotaxis, moves toward attractants and away from repellents with the help of transmembrane chemoreceptors known as methyl-accepting chemotaxis proteins (MCPs) that form clusters at cell poles.^14^^,^^16^ When the periplasmic domain of MCPs binds the corresponding chemoeffectors (e.g., attractants),^17^^,^^18^ conformational changes are triggered in the receptors.^19^^,^^20^^,^^21^^,^^22^ This leads to suppression of the activity of the associated histidine kinase (CheA), and consequently a decrease in the level of phosphorylation of the diffusible response regulator CheY.^23^ Phosphorylated CheY (called CheY-P) binds to the switch complex of the flagellar motor and changes the probability of the motor rotating counterclockwise (CCW) or clockwise (CW),^24^ and accordingly the probability of cell run and tumble.^25^^,^^26^ CheY-P can be dephosphorylated by the phosphatase CheZ.^26^

Adaptation of the chemotaxis network is accomplished by the competitive action of the methyltransferase CheR and methylesterase CheB, which methylates (CheR) or demethylates (CheB) the receptors on several specific sites and increases or decreases the receptor-kinase activity, respectively.^16^^,^^27^^,^^28^ The methylation and demethylation rates depend on the kinase activity, which forms a negative feedback mechanism for adaptation. There are four or five modifiable sites encoded in each chemoreceptor monomer that can be methylated or demethylated. Biochemical experiments showed that a pentapeptide (NEWTF or NWESF) at the extreme C-terminus of the receptor cytoplasmic domain provides the docking site for CheR and CheB that allows them to localize to the receptor clusters.^29^^,^^30^ The C-terminal pentapeptide is only encoded in the two major receptors, Tar and Tsr. Studies with electron paramagnetic resonance spectroscopy showed that there is a flexible linker between the conserved pentapeptide and the coiled-coil receptor body.^31^ The length of the linker is approximately 30 residues, and is variable among different kinds of chemoreceptors. Linker-truncation studies demonstrated that the linker has a significant impact on the function of chemoreceptors.^32^ Measurements *in vitro* showed that the tethered CheR and CheB can act on an assistance neighborhood of five to seven nearby receptors, which is necessary for precise adaptation in a model of receptor clusters composed of different types of receptors.^33^^,^^34^

High precision of adaptation due to the negative feedback mechanism is a general consensus in bacterial chemotaxis. A corollary of precise adaptation is the independence of the adaptation kinetics (methylation/demethylation rates) on the methylation level. Nevertheless, the detailed kinetics of receptor methylation and demethylation remain unclear. Previous experiments established sequentiality among the different methylation sites on the receptor.^35^^,^^36^^,^^37^^,^^38^ A recent study suggested that sequential modification of multiple methylation sites is critical for perfect adaptation.^39^ Furthermore, imprecise adaptation to high concentrations of serine (a ligand sensed by the receptor Tsr), and high concentrations of aspartate or its non-metabolizable analogue α-methyl-DL-aspartate (MeAsp) that are sensed by the receptor Tar, have been reported, which were generally thought to be caused by saturation of available modification sites on the receptors.^40^^,^^41^^,^^42^^,^^43^^,^^44^ Here, we studied the adaptation kinetics of Tar-only strains by examining the step responses to various concentrations of MeAsp. We found a sharp decrease in the methylation rate with increasing methylation level. We suspected that this phenomenon is related to the diffusion of flexible tethers and explained the adaptation kinetics with a mechanism of tethered particle motion (TPM) of CheR. We performed additional experiments with different lengths of linkers for tethering CheR, providing further evidence for the mechanism of TPM for the methylation kinetics. The TPM resulted in a near-exponential distribution of CheR concentration along the sequential modifiable sites, so that CheR molecules encounter the preceding sites much more frequently than the ensuing sites. An analytical model for sequential multisite methylation/demethylation showed that this avoids saturating the methylation sites, increases chemotactic sensitivity, and also ensures robustness of methylation to fluctuations in kinase activity and CheR/CheB expression.

The Results section below is organized as follows. First, we observed a strong dependence of the methylation rate on the methylation level in the Tar-only strain through experimental measurements, and measured the adaptation kinetics over a wide range of methylation levels. Second, these experimental results could not be explained by the model of scarce methylation/demethylation sites. Third, we successfully accounted for our experimental results by introducing the TPM model of CheR, and experiments with different linker lengths further validated our model. Finally, an analytical model was used to study the physiological significance of the TPM of CheR.

## Results

### The methylation rate decreased sharply with increasing methylation level in the Tar-only strain

Sensing and adapting of *E. coli* to stimuli could be described by a coarse-grained model of the chemotaxis signaling pathway.^45^ The kinase activity of a Monod-Wyman-Changeux (MWC) cluster containing *N* receptors was determined by

(Equation 1) $$a=\frac{1}{1+\exp\left(N\left(f_{m}+f_{L}\right)\right)}\text{,}$$

where a denotes the receptor-kinase activity, $f_{m}=\alpha \left(m-m_{0}\right)$ and $f_{L}=\ln\frac{1+L/K_{off}}{1+L/K_{on}}$ represent the methylation-dependent and ligand-dependent free energy, respectively, L is the ligand concentration, and m denotes the methylation level whose evolution follows the differential equation:

(Equation 2) $$\frac{dm}{dt}=G\left(a,m\right)\text{,}$$

where perfect adaptation requires that *G*(*a, m*) is a function of *a* only and is independent of *m*. The detailed values of the parameters we used here are listed in the STAR Methods.

**Figure 1:**
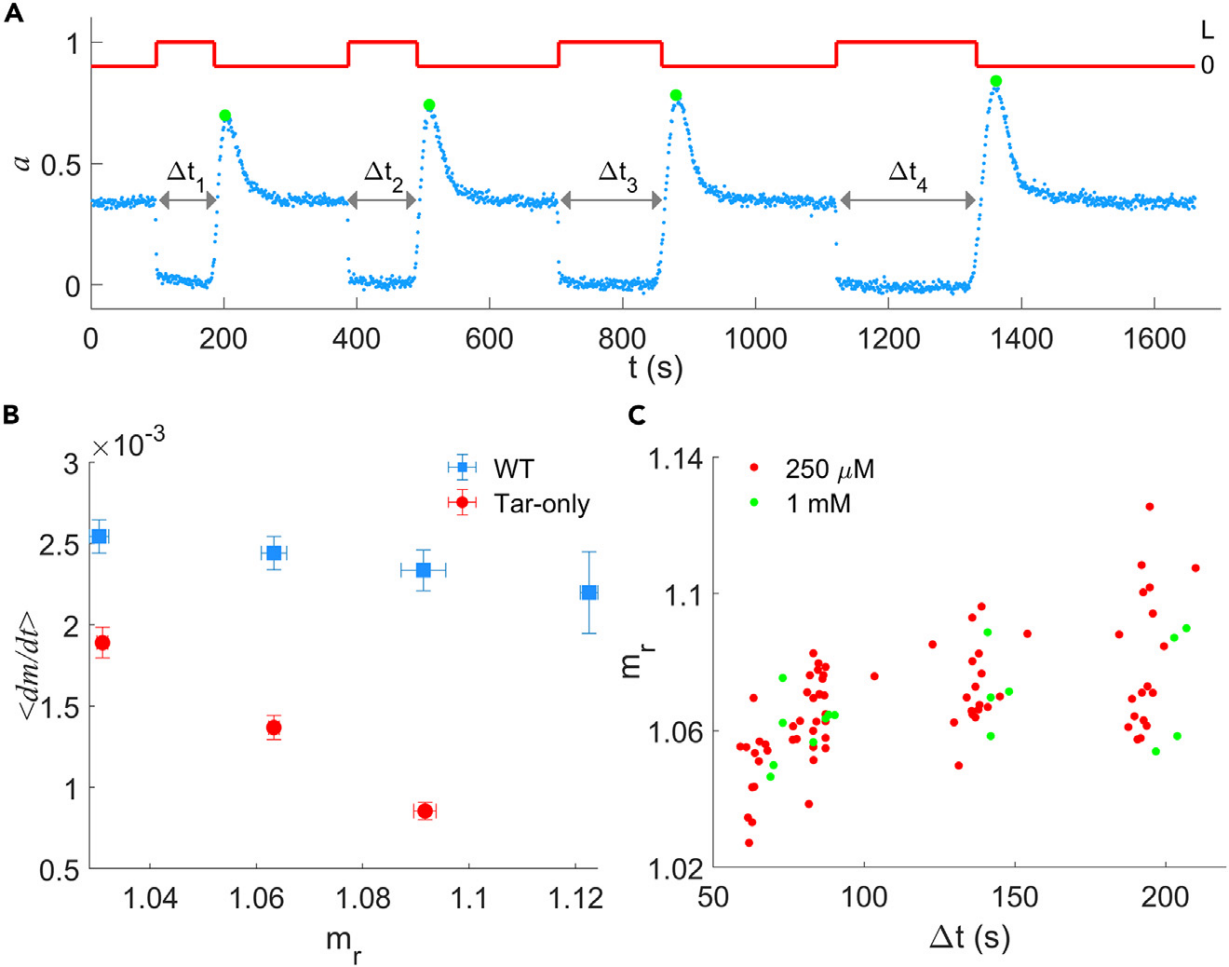
Measurements of the relationship between the methylation rate and the methylation level (A) Typical trace of kinase activity response to saturated stimulus for the Tar-only strain via zero-activity assay. Blue dots are experimental data, while the red solid line indicates the concentration change of a saturated stimulus (250 μM MeAsp). (B) The average methylation rate ⟨dm/dt⟩ as a function of the methylation level $m_{r}$ for the wild-type (blue squares) and Tar-only (red dots) strains under a saturating stimulus of 250 μM MeAsp. Errors denote SEM. (C) The methylation level as a function of the duration time at zero activity of the Tar-only strain with saturated stimuli of 250 μM MeAsp (red dots) or 1 mM MeAsp (green dots). Each dot represents one measurement.

To test whether the net methylation rate (methylation rate minus the demethylation rate) *G*(*a, m*) is independent of *m*, we tried to measure the methylation level after inducing the kinase activity to zero for different durations via stepwise addition of a saturated stimulus. As shown in Figure 1A, we followed the receptor kinase activity *in vivo* by monitoring the FRET signal between the response regulator CheY and its phosphatase CheZ, which were fused with eYFP and eCFP, respectively. The saturated stimulus was added for $\Delta t_{1}$ or $\Delta t_{2}$ (the magnitudes of $\Delta t_{1}$ and $\Delta t_{2}$ are in random order), during which the kinase activity stayed zero. We recorded the corresponding activities $a_{1}$ or $a_{2}$ immediately after removal of the stimulus, from which we could compute the methylation level $m_{1}$ or $m_{2}$ using Equation 1.

We recorded the relationship between kinase activity after removal of 250 μM MeAsp ($a_{r}$) and methylation time (Δt) for both the wild-type strain (HCB1288-pVS88) and the mutant strain expressing only Tar receptors (HCB1414-pLC113-pVS88). The $a_{r}$ for Δt=0 represents the pre-stimulus kinase activity. We calculated the corresponding methylation level $m_{r}$ with Equation 1, and evaluated the net average methylation rate $\left\langle dm/dt\right\rangle =\left(m_{r}-m_{t_{m}=0}\right)/\Delta t$, where $m_{t_{m}=0}$ denotes the pre-stimulus methylation level. The relations between ⟨dm/dt⟩ and $m_{r}$ for both strains are shown in Figure 1B. The errors denote SEM. The net average methylation rate decreases sharply with the methylation level for the Tar-only mutant, whereas it remains nearly constant for the wild-type strain. To further ensure that our measurements were not influenced by possible alterations in cellular state, we calculated and compared the ⟨dm/dt⟩ for two different values of Δt, both before and after a 20-min period. As shown in Figure S1, the observed effects are not a result of alterations in cellular state caused by differences in recording time or other possible factors.

The higher net average methylation rate of the wild-type strain may result from the adaptational “assistance neighborhoods” consisting of other types of receptors, which provide more modifiable sites for the tethered CheR to Tar.^33^ We also performed the measurement with another concentration of saturated stimuli (1 mM MeAsp). The relations between $m_{r}$ and Δt under the two different concentrations of MeAsp are shown in Figure 1C for the Tar-only strain. They follow a similar trend, so the net methylation rate is not affected by the concentration of the saturating stimuli. Given that the entire adaptation was performed in the inactive state of Tar receptors (a=0), in which the methylation of receptors by CheR was dominant, the change in methylation level resulted mainly from methylation by CheR. All these results demonstrated a clear dependence of the methylation rate on the methylation level, indicating imprecise adaptation. More traces of step responses for the Tar-only strains are shown in Figure S2.

### Measurement of the relationship between adaptation kinetics and methylation level via the steady-state adapted activity

We found a strong dependence of the methylation rate on the methylation level for the Tar-only strain. However, the measured variation range of methylation levels is small due to technical limitations. We thus sought a new way to measure the adaptation kinetics over a wider range of methylation levels.

Using a canonical model of the adaptation dynamics where CheR and CheB only act on inactive or active receptors, respectively,^46^^,^^47^

(Equation 3) $$\frac{dm}{dt}={G\left(a,m\right)=k}_{R}\left(1-a\right)-k_{B}a\text{,}$$

where $k_{R}$ and $k_{B}$ represent the methylation and demethylation rates catalyzed by CheR and CheB, respectively. The adapted activity *a*∗ at the steady state (dm/dt=0) would satisfy

(Equation 4) $$\frac{a^{*}}{1-a^{*}}=\frac{k_{R}}{k_{B}}=F\left(m\right)\text{,}$$

**Figure 2:**
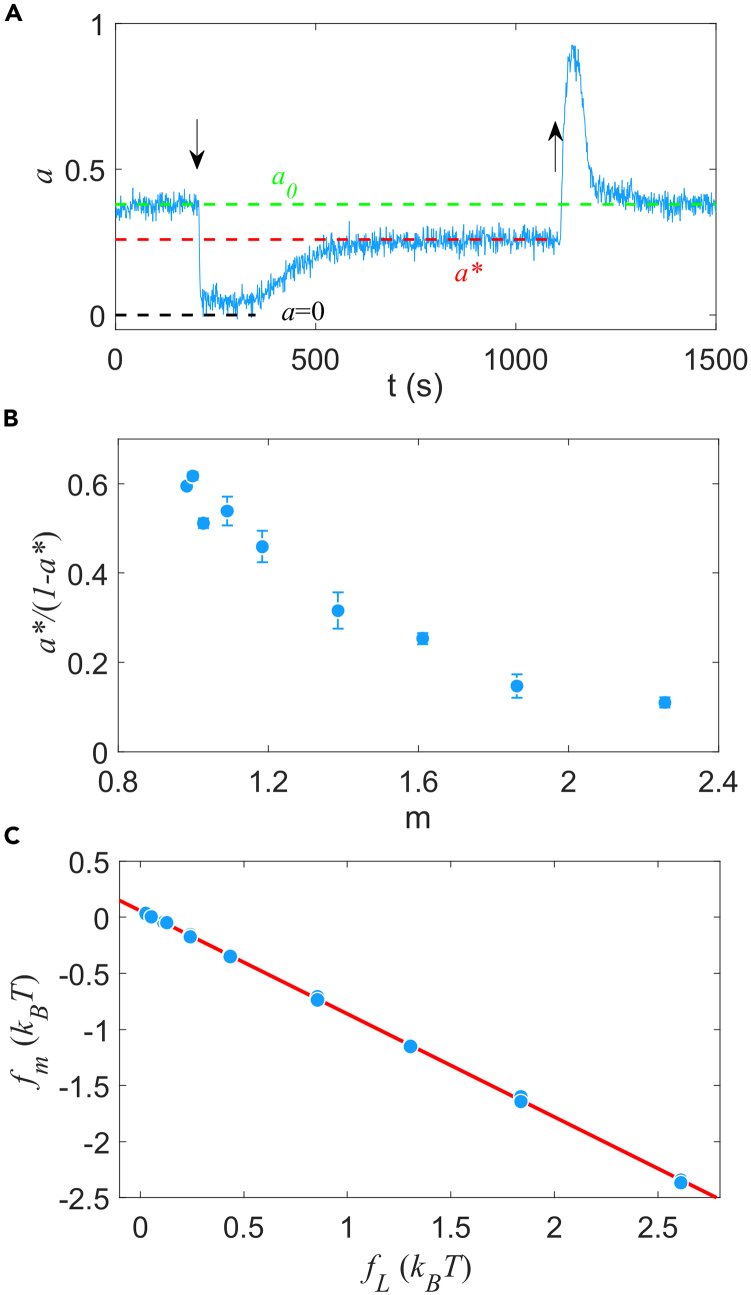
The response of the Tar-only strain to stepwise addition of MeAsp (A) A typical step response of kinase activity to 25 μM MeAsp for the Tar-only strain. The blue solid line denotes the signal of kinase activity. The green, red, and black dashed lines represent the pre-stimuli activity *a*_0_, the adapted activity *a∗* and zero activity, respectively. The down and up arrows indicate the moment of adding and removing MeAsp, respectively. (B) The relation between *a*∗/(1-*a*∗) and the methylation level calculated from our data of the Tar-only strain. Errors are SD. (C) The relation between ligand-dependent (*f*_*L*_) and methylation-dependent (*f*_*m*_) free energy of the Tar-only strain. Experimental data (blue dots) are fitted with a linear function (red line). Each data point represents the result of one step-response experiment.

Thus, we could obtain the relation between $k_{R}/k_{B}$ and the adapted methylation level m by measuring the adapted activity *a*∗ after stepwise addition of different concentrations of MeAsp (corresponding to different values of *m*). A typical step response of kinase activity to 25 μM MeAsp for the Tar-only strain is shown in Figure 2A. The chemical was added at *t* = 200 s and removed at *t* = 1100 s. The adapted activity *a*∗ is defined as the average of steady-state kinase activity before removing MeAsp. The cells exhibited imprecise adaptation, which means that the adapted activity *a*∗ is below the pre-stimuli activity *a*_0_. Step responses to different concentrations of MeAsp were measured, and the adapted activity *a*∗ and the corresponding MeAsp concentration *L* were recorded (see STAR Methods for details), from which the corresponding adapted methylation level *m* was obtained by using Equation 1.

The relation between *a*∗/(1-*a*∗) and *m* measured for the Tar-only strain is shown in Figure 2B, exhibiting a sharp dependence of *a*∗/(1-*a*∗), and thus $k_{R}/k_{B}$, on *m*. We tried to explain it with the model of scarce methylation/demethylation sites, in which *k*_*R*_ and *k*_*B*_ were assumed to be dependent on the methylation level by multiplying by a factor of *M*/(*M* + *M*_*sat*_), where *M* is the total number of available sites for methylation or demethylation and *M*_*sat*_ is a constant.^41^^,^^43^ However, this model could not fit our data well with reasonable fitting parameters (Figure S3), suggesting that the saturation of available methylation/demethylation sites may not be the main cause of the strong dependence we found here.

As shown in Figure 2C, we also found a surprising linear relationship between the adapted $f_{m}$ and $f_{L}$ with a slope of −0.920 ± 0.003 (perfect adaptation would require a slope of −1.0), indicating that the imprecise adaptation for the Tar-only strain is manifested to a similar extent over the whole range of MeAsp concentrations. This further asked for a new mechanism of imprecise adaptation, which we sought to establish below.

### Strong dependence of the methylation rate on the methylation level due to the TPM of CheR

CheR and CheB (CheB-P) molecules are tethered to the receptors via an unordered polypeptide chain at the C-terminus of the receptors.^31^^,^^48^ The binding site is a pentapeptide (NWETF) at the extreme C-terminus of the polypeptide. Isothermal titration calorimetry (ITC) experiments showed that the affinity of binding to the pentapeptide is approximately 2 μM and 160 μM for CheR and CheB, respectively.^30^^,^^49^^,^^50^ It was proposed that binding to the pentapeptide could increase the local CheR concentration around the methylation sites, whereas the interaction between CheB and the pentapeptide is too weak to have an obvious effect on local concentration.^8^^,^^33^^,^^49^ Thus, we considered the effect of TPM for CheR only.

**Figure 3:**
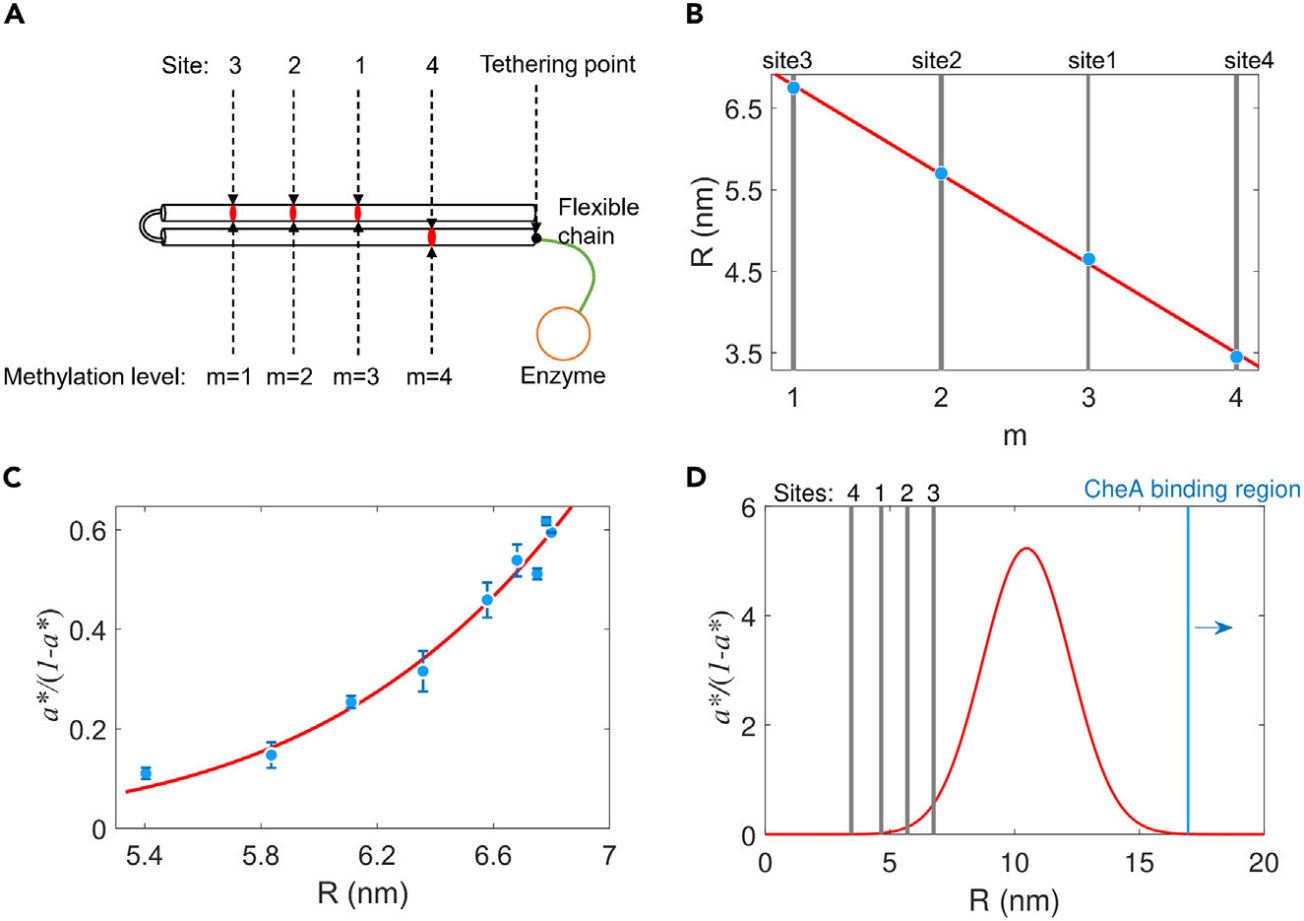
The adaptation kinetics due to tethered particle motion of the methylation enzyme CheR (A) The simplified cartoon of a Tar monomer showing the modifiable portion along with the tethered enzyme. Black cylinders represent two parallel α-helices encoded with four modifiable sites (red dots). The green curve indicates the C-terminal flexible chain with 34 residues. The large red circle connected to the chain denotes the tethered enzyme. (B) The relation between the distance (*R*) of each modifiable site from the tethering point (black dot in A) and the methylation level (*m*). The gray lines denote the modifiable sites. The red line is the fit with a linear function. (C) Fitting of the tethered particle model to the experimental data measured with the Tar-only strain. The blue dots are the experimental data. The red line is the fit. Errors are SD. (D) Complete fitting curve (red line) of Figure 3C along with positions of the modifiable sites (gray lines) and CheA binding region (blue line denotes position of residue 401).

According to previous studies of the receptor structure, we can produce a simplified sketch for the arrangement of the methylation/demethylation sites and the CheR tethering site for the Tar receptor.^51^^,^^52^ As shown in Figure 3A, four modification sites lie on two parallel α-helices, with sites #1 to #3 on the same helix and site #4 on the other. The CheR enzyme is tethered to the C-terminal helix via a flexible polypeptide chain. Biochemical experiments suggested a sequential order of methylation: sites #3 → #2 → #1 → #4,^37^^,^^38^ and recent theoretical analysis suggested that the methylation process for the four sites is mostly sequential.^39^ Therefore, we treat the methylation levels *m* = 1, 2, 3, and 4 corresponding to the situations immediately after sites #3, #2, #1, and #4 are methylated, respectively. We can estimate the distance of each site from the tethering point by a translation of 0.15 nm along the helical axis for each residue in an α-helix. Hence, we related the methylation level *m* to the distance *R* from the tethering point, as plotted in Figure 3B. The red solid line denotes the result of fitting with a linear function *R* = *k* × *m* + *R*_0_, with *k* = −1.10 ± 0.06 and *R*_0_ = 7.86 ± 0.16. Using this linear relation, we can convert the horizontal axis (*m*) of Figure 2B to *R*, as denoted in Figure 3C as blue dots, showing *a*∗/(1−*a*∗) as a function of *R*. Equivalently, we converted the function *F*(*m*) in Equation 4 to a function of *R*,

(Equation 5) $$\frac{a^{*}}{1-a^{*}}=\frac{k_{R}}{k_{B}}=g\left(R\right)\text{,}$$

We treated the diffusion of tethered CheR as a TPM, modeling the CheR molecule as a ball that is tethered to a wall (the receptor) via a flexible peptide chain of 34 residues. The probability density function of the distance between two ends of a freely joined chain is described by a Gaussian function^53^:

(Equation 6) $$P\left(\vec{R}\right)=C\times \exp\left(\frac{{-3\vec{R}}^{2}}{2L_{total}b}\right)\text{,}$$

where $\vec{R}$ is the vector from the tethering point to the other end of the chain, *C* is a normalization constant, $L_{total}$ denotes the contour length of the chain, and *b* is the Kuhn length, which is equal to twice the persistence length (0.38 nm) as measured by fitting with the worm-like-chain model.^54^ The contour length of the chain $L_{total}$ equals 12.24 nm, calculated from the peptide bond length of 0.36 nm and the number of residues of 34 between the tethering point and binding pentapeptide.^55^

Due to the volume-exclusion effects,^56^^,^^57^^,^^58^ the ball is excluded from the region of space occupied by the chain and the wall, and the peak of the probability distribution for the ball position is translated by a distance that depends on both the chain contour length and the ball size.^56^ Hence, we use a translated Gaussian distribution to describe the positional distribution of CheR:

(Equation 7) $$P_{CheR}=C\times \exp\left(\frac{-3{\left(R-x_{0}\right)}^{2}}{2L_{total}b}\right)\text{,}$$

where $x_{0}$ denotes the translation that is affected by the size of the CheR molecule and the length of the peptide chain. As the methylation rate $k_{R}$ is proportional to the concentration of CheR, the function g(R) in Equation 5 is proportional to $P_{CheR}$. Therefore, we can rewrite Equation 5 as

(Equation 8) $$\frac{a^{*}}{1-a^{*}}=c\times \exp\left(\frac{-3{\left(R-x_{0}\right)}^{2}}{2L_{total}b}\right)\text{,}$$

where *c* is a proportional constant. Equation 8 can fit well to our data, as shown in Figure 3C, extracting a value of *x*_0_ of 10.5 ± 0.4 nm. As shown in Figure 3D, the position of the Gaussian peak is between the covalent modification region and CheA binding region^59^ of Tar receptors, suggesting that the translation of Gaussian distribution is a trade-off between increasing the local concentration of the modification enzyme and ensuring CheB phosphorylation by CheA.

To show that our analysis did not depend on the specific model of adaptation dynamics, we also tried the adaptation model with a much stronger sensitivity of the demethylation rate on activity, which was proposed to explain the asymmetry in the time courses of stepwise addition and removal of attractant^43^:

(Equation 9) $$\frac{dm}{dt}=k_{R}\left(1-a\right)-k_{B}a^{3}\text{,}$$

where $k_{R}$ and $k_{B}$ represent the methylation and demethylation rates catalyzed by CheR and CheB, respectively. For the steady state,

(Equation 10) $$\frac{{a^{*}}^{3}}{1-a^{*}}=c\times \exp\left(\frac{-3{\left(R-x_{0}\right)}^{2}}{2L_{total}b}\right)\text{,}$$

where the parameters share the same definitions as Equation 8. Equation 10 can also fit well to our data, as shown in Figure S4. Thus, interpretation of our experimental data with the TPM model is independent of the specific adaptation model. Furthermore, we attempted to extend our model to the Tsr and Trg receptors. By measuring the adaptive behavior of Tsr-only strains in response to different concentrations of 2-aminoisobutyric acid (a non-metabolic analogue of serine^60^), we found that our model could also fit the results for Tsr receptors well (Figure S5). In addition, based on the structural analysis of Trg, we demonstrated that the dependence of the methylation rate on the methylation level could also be applied to chemoreceptors lacking a CheR-binding pentapeptide (see Figure S6 and supplemental information). However, this dependence cannot be measured directly because additional Tar or Tsr receptors need to be expressed to provide the tethered CheR, which will provide more modifiable sites and weaken the dependence of the methylation rate on the methylation level, similar to that found for the wild-type strain.

Combining the TPM model with Equation 1 leads to a relationship between $f_{L}$ and $f_{m}$: $f_{L}=0.006f_{m}^{2}-1.07f_{m}+f_{0}$, where $f_{0}$ is a constant. The relationship between $f_{L}$ and $f_{m}$ is actually quadratic, but the second-order term is much smaller than the first-order term for the range of $f_{m}$ we observed here and thus is negligible. This results in a linear relationship between $f_{m}$ and $f_{L}$ with a slope of 1/1.07 ≈ 0.93, consistent with our measurement.

### Experiments with different lengths of the tethering peptide chain further validate the TPM model

To further validate the tethered particle model for the adaptation kinetics, we changed the contour length ($L_{total}$) of the flexible chain in Tar, and performed step-response experiments to study imprecision in adaptation for the Tar-only strain. We made three plasmids that express mutant Tar receptors with different lengths of the unstructured peptide chain under the control of an arabinose-inducible promoter. Plasmids pCZ1, pCZ2 and pCZ4 were used to express Tar containing a flexible chain with 29, 22 and 46 residues, respectively (see Table 1).

**Table 1:** The amino acid sequence of the tethering chain

Name	amino acid sequence of chain (containing the pentapeptide)
pLC113 wild-type Tar (34 aa chain)	LAASPLTNKPQTPSRPASEQPPAQPRLRIAEQDP **NWETF**
pCZ1 mutant Tar (29 aa chain)	LAASPLTNKPQTPSRPASEQPPAQPRLRI --------- **NWETF**
pCZ2 mutant Tar (22 aa chain)	LAASPLTNKPQTPSRPASEQPP ---------------------- **NWETF**
pCZ4 mutant Tar (46 aa chain)	LAASPLTNKPQTPSRPASEQPPAQPRLRIAEQDP APRKMAVADSEE **NWETF**
Wild-type Tsr	IQQQQRETSAVVKTVTPAAPRKMAVADSEE**NWETF**

**Figure 4:**
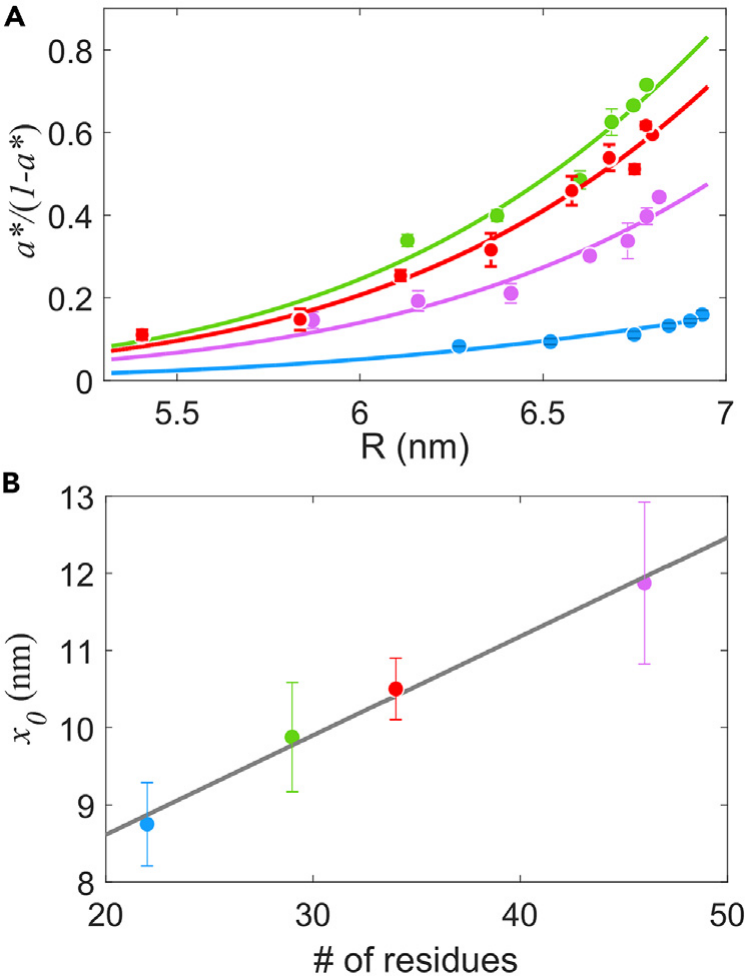
Experiments with various lengths of the C-terminal flexible chain in the Tar receptor (A) Fitting of the tethered particle model to experimental data with four Tar-only strains expressing different Tar constructs. Results from the strain with the wild-type Tar receptor containing a flexible chain with 34 residues are indicated by red dots. Results from the strains with the mutant Tar receptors containing a flexible chain with 29, 22 and 46 residues are represented by green, blue and purple dots, respectively. (B) The relation between the fitted translational distance *x*_*0*_ (extracted from A with SD) and the length of the flexible chain. The gray line is the fit with a linear function.

We measured the dose-response curve and performed the step-response experiments for each Tar mutant as before. The results of *a*∗/(1−*a*∗) as a function of *R* for different lengths of the tethering chain are shown in Figure 4A. Equation 8 can fit well to the data using different values of *L*_*total*_ calculated from the different numbers of residues in the flexible chain, extracting different values of *x*_0_ for the different constructs of Tar. We plotted *x*_0_ as a function of the number of residues in the flexible chain (Figure 4B), which can be fit with a linear function with a slope of 0.128 ± 0.048 nm/aa and a *y* intercept of 6.0 ± 0.6 nm. The slope is consistent with the previous Brownian dynamics simulation of the ball-and-chain model, in which a most probable end-to-end distance of 4.1 nm was obtained for a polypeptide of 32 residues if the ball size was large enough (>2.0 nm) to induce the volume-exclusion effects.^56^ The *y* intercept of 6 nm is consistent with the size of the CheR molecule or the distance between the catalytic and pentapeptide-binding sites on CheR measured by electron microscopy.^61^ We also used Equation 10 to fit the data for all strains, as shown in Figure S7A. Each of them exhibits a good fit, and the relation between *x*_0_ and the number of residues in the flexible chain can also be fitted with a linear function (see Figure S7B). The fitted slope and *y* intercept are 0.233 ± 0.100 nm/aa and 7.4 ± 3.4 nm, respectively. This further confirms the validity of the TPM model irrespective of the specific model of adaptation dynamics we used.

### Physiological significance of the encounter-rate matching of sequential modifiable sites

The TPM resulted in a near-exponential distribution of CheR concentration along the sequential modifiable sites, such that the CheR molecules encounter the preceding sites much more frequently than the ensuing sites. To elucidate the physical significance of this effect, we constructed a multisite modification model for the sequential methylation/demethylation of MCP, similar to the multisite phosphorylation model in cell signaling.^62^^,^^63^^,^^64^

**Figure 5:**
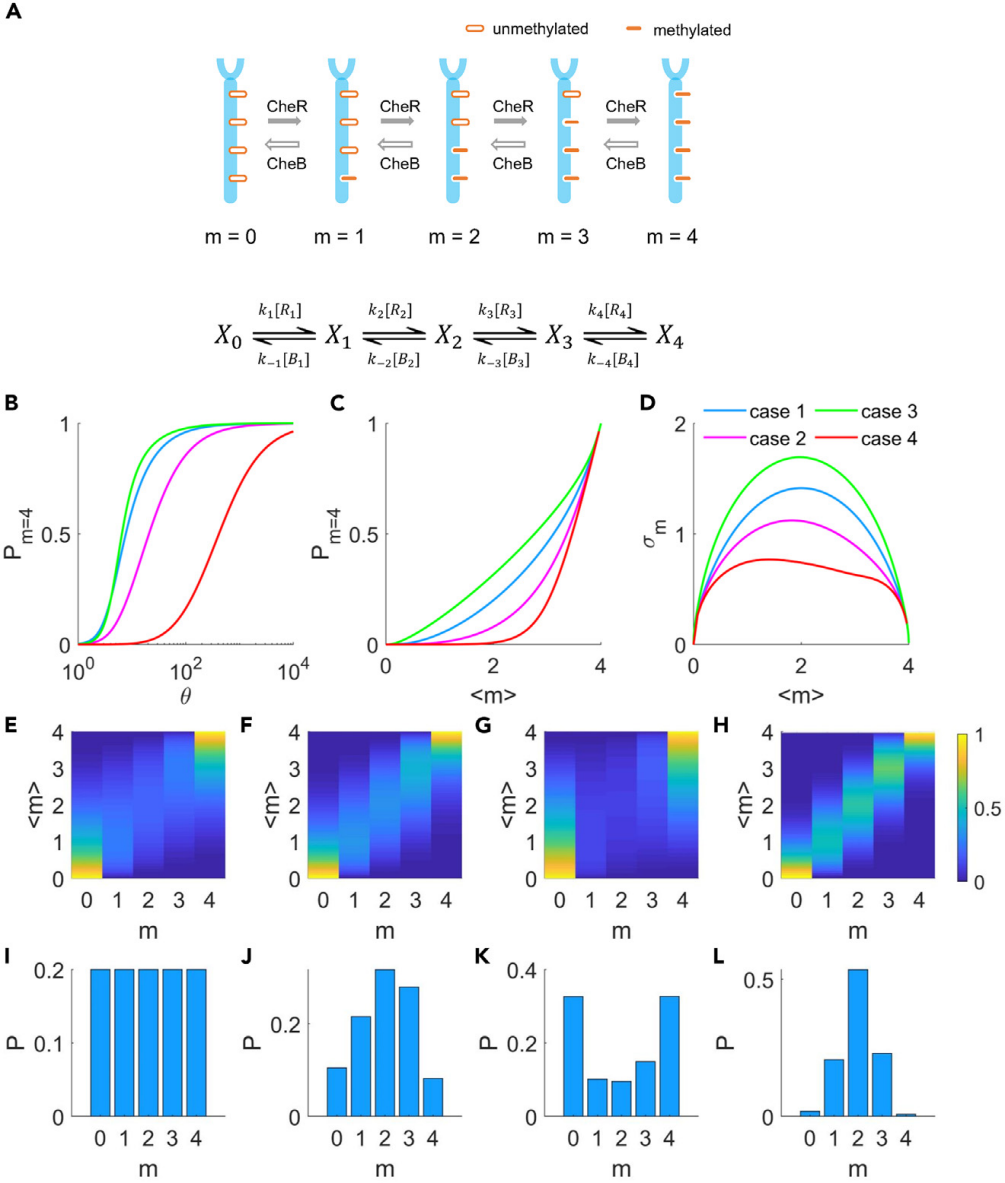
The analytical model of multisite catalytic reaction for chemoreceptors (A) Schematic illustration of methylation and demethylation dynamics for a receptor with four modifiable sites. (B) The relation between the probability of all sites (four) being methylated and the dose $\theta =K_{eq}\left[R_{tot}\right]/\left[B_{tot}\right]$. (C) The relation between the probability of saturated methylation sites and the average methylation level for the four cases. (D) The standard deviation of the methylation level $\sigma_{m}$ at different mean methylation levels ⟨m⟩. In B–D, four cases of CheR encounter probability settings for the four sites are shown. Case 1: P_1_ = P_2_ = P_3_ = P_4_ = 0.25, blue line; Case 2: P_1_ = 7/16, P_2_ = 5/16, P_3_ = 3/16, P_4_ = 1/16, pink line; Case 3: P_1_ = 1/16, P_2_ = 3/16, P_3_ = 5/16, P_4_ = 7/16, green line; Case 4: P_1_ = 0.7809, P_2_ = 0.1856, P_3_ = 0.0309, P_4_ = 0.0026, red line. (E–H) The evolution of $P_{m=0,1,2,3,4}$ with ⟨m⟩ for cases 1–4 respectively. The color map indicates the probability. (I–L) The probability distributions of methylation levels for cases 1–4, respectively, when the average methylation level is 2.

The methyltransferase CheR and methylesterase CheB (or CheB-P) could act on receptor *X* with four sequential modifiable sites. For a single encounter, at most one modification (methylation or demethylation) was thought to take place before the enzyme and substrate molecules part company. The chain of enzymatic reactions is shown in Figure 5A. The rate equations are

$$\frac{d\left[X_{0}\right]}{dt}=k_{-1}\left[B_{1}\right]\left[X_{1}\right]-k_{1}\left[R_{1}\right]\left[X_{0}\right]\text{,}$$

$$\frac{d\left[X_{i}\right]}{dt}={k_{-\left(i+1\right)}\left[B_{i+1}\right]\left[X_{i+1}\right]-k_{i+1}\left[R_{i+1}\right]\left[X_{i}\right]+k}_{i}\left[R_{i}\right]\left[X_{i-1}\right]-k_{-i}\left[B_{i}\right]\left[X_{i}\right]\text{,}$$

where *i* = 1 to 3

$$\frac{d\left[X_{4}\right]}{dt}=k_{4}\left[R_{4}\right]\left[X_{3}\right]-k_{-4}\left[B_{4}\right]\left[X_{4}\right]\text{,}$$

where $\left[X_{i}\right]$ is the concentration of receptors with a methylation level of *i*. $k_{i}$ and $k_{-i}$ represent the reaction rate constants of methylation and demethylation for site *i*, respectively, which include the dependence on the receptor activity with factors of (1−*a*) and *a*, respectively. For a global adaptation (GA) scheme,^65^ a=⟨a⟩ is the global averaged activity that is independent of *i* for a specific receptor, and is determined by the average methylation level ⟨m⟩. To study the effect of site-dependent concentrations of enzymes more clearly, we chose this model in the main text. The analysis using the more complex local adaptation (LA) scheme,^65^ in which *a* is the activity of a specific receptor and is dependent on *i*, is presented in the supplemental information and Figure S10, which leads to similar conclusions to those derived from the GA model. $\left[R_{i}\right]$ and $\left[B_{i}\right]$ denote the concentrations of CheR and CheB-P at site *i*, respectively. At steady state when the adaptation was complete, the rate of change for the concentration of each methylated form was zero. Thus,

(Equation 11) $$k_{i}\left[R_{i}\right]\left[X_{i-1}\right]=k_{-i}\left[B_{i}\right]\left[X_{i}\right]\text{,}$$

where i=1,2,3,4. Then,

$$\left[X_{i}\right]=\frac{k_{i}\left[R_{i}\right]}{k_{-i}\left[B_{i}\right]}\left[X_{i-1}\right]=\frac{k_{i}\left[R_{i}\right]}{k_{-i}\left[B_{i}\right]}\frac{k_{i-1}\left[R_{i-1}\right]}{k_{-\left(i-1\right)}\left[B_{i-1}\right]}\left[X_{i-2}\right]=\left(\prod_{j=1}^{i}\frac{k_{j}}{k_{-j}}\frac{\left[R_{j}\right]}{\left[B_{j}\right]}\right)\left[X_{0}\right]=\left(\prod_{j=1}^{i}K_{eq}^{j}u_{j}\right)\left[X_{0}\right]$$

where $K_{eq}^{j}=k_{j}/k_{-j}$ and $u_{j}=\left[R_{j}\right]/\left[B_{j}\right]$. We can then calculate the probability of receptors with a methylation level of *i* (*P*_*m=i*_); specifically, the probability of receptors with all four sites methylated is

(Equation 12) $$P_{m=4}=\frac{\left[X_{4}\right]}{\left[X_{0}\right]+\left[X_{1}\right]+\left[X_{2}\right]+\left[X_{3}\right]+\left[X_{4}\right]}=\frac{\prod_{i=1}^{4}K_{eq}^{i}u_{i}}{1+K_{eq}^{1}u_{1}+\prod_{i=1}^{2}K_{eq}^{i}u_{i}+\prod_{i=1}^{3}K_{eq}^{i}u_{i}+\prod_{i=1}^{4}K_{eq}^{i}u_{i}}$$

Based on the GA scheme, we assumed that [*B*_*i*_], *k*_*i*_, and *k*_*-i*_ were independent of site *i*: $\left[B_{i}\right]=\left[B_{tot}\right]$ and $K_{eq}^{i}=K_{eq}$, whereas the dependence of CheR concentration on site *i* was explicitly included: $\left[R_{i}\right]=\left[R_{tot}\right]*P_{i}$. $\left[B_{tot}\right]$ and $\left[R_{tot}\right]$ represent the concentrations of CheB-P and CheR in a cell, respectively. $P_{i}$ denotes the proportion of $\left[R_{tot}\right]$ at the ith site, and $\sum_{i=1}^{4}P_{i}=1$. Rearranging Equation 12 leads to

(Equation 13) $$P_{m=4}\left(\theta \right)=\frac{\theta^{4}P_{1}P_{2}P_{3}P_{4}}{1+\theta P_{1}+\theta^{2}P_{1}P_{2}+\theta^{3}P_{1}P_{2}P_{3}+\theta^{4}P_{1}P_{2}P_{3}P_{4}}\text{,}$$

where $\theta =K_{eq}\left[R_{tot}\right]/\left[B_{tot}\right]$ and is related to the kinase activity and CheR/CheB expression levels. We considered the methylation dynamics under four different cases, where Case 4 used the probability values from our measurements.

Case 1: P_1_ = P_2_ = P_3_ = P_4_ = 0.25 (constant probability).

Case 2: P_1_ = 7/16, P_2_ = 5/16, P_3_ = 3/16, P_4_ = 1/16 (linearly decreasing probability).

Case 3: P_1_ = 1/16, P_2_ = 3/16, P_3_ = 5/16, P_4_ = 7/16 (linearly increasing probability).

Case 4: P_1_ = 0.7809, P_2_ = 0.1856, P_3_ = 0.0309, P_4_ = 0.0026 (matched probability).

The relation between $P_{m=4}$ and θ for all four cases is plotted in Figure 5B. All of them exhibit a sigmoidal shape. We extracted *θ*_*0.1*_ and *θ*_*0.9*_, the values of *θ* required to reach 10% and 90% for *P*_*m=4*_, respectively. From them, the Hill coefficient *H* can be extracted. As *m* = 4 denotes the unwanted situation with methylation sites saturated, ideally *H* should be small to allow robustness of methylation to the fluctuations in *θ*, and *θ*_*0.1*_ should be large to avoid saturation of the methylation sites. The values of *H* we extracted for the four cases are 1.7, 1.3, 2.2, and 1.1, and the values of *θ*_*0.1*_ are 3.0, 5.3, 3.2, and 65.6 for cases 1 to 4, respectively, both demonstrating that Case 4 is the optimal strategy.

We calculated the average methylation level and the standard deviation in the distribution of methylation levels at each value of *θ*: $\left\langle m\right\rangle =\sum_{i=0}^{4}i*P_{m=i}$ and $\sigma_{m}=\sqrt{\sum_{i=0}^{4}P_{m=i}*{\left(i-\left\langle m\right\rangle \right)}^{2}}$. We then plot *P*_*m=4*_ as a function of ⟨m⟩, as shown in Figure 5C for the four cases. Clearly, the threshold ⟨m⟩ for saturating the methylation sites is the highest for Case 4. To quantify this, we extracted ${\left\langle m\right\rangle }_{0.1}$, which was the value of ⟨m⟩ when *P*_*m=4*_ reached 10%, leading to 1.4, 2.2, 0.9, and 2.8 for cases 1 to 4, respectively. *σ*_*m*_ as a function of ⟨m⟩ for the four cases are shown in Figure 5D, and Case 4 exhibits the lowest and a nearly constant standard deviation for 0<⟨m⟩<4 among all cases. A small *σ*_*m*_ indicates a more uniform methylation level over the receptor population, again avoiding saturation of the methylation sites for some of the receptors. This can be seen more clearly in the color maps of Figures 5E–5H, where $P_{m=0,1,2,3,4}$ for different values of ⟨m⟩ for cases 1 to 4 are shown (E to H represent cases 1 to 4, respectively). Case 4 exhibits the optimal situation among all cases: for each ⟨m⟩, the probability distribution of methylation levels centers around the value of *m* equaling ⟨m⟩, resulting in a near-perfect correlation in the color map, thereby reducing variation in the methylation level among the receptor population and avoiding premature saturation of methylation sites. As a concrete example, we plotted $P_{m=0,1,2,3,4}$ at ⟨m⟩=2, as shown in Figures 5I–5L for cases 1 to 4, respectively, demonstrating a sharp peak at *m* = 2 with near-zero probability at *m* = 0 and 4 for Case 4, compared to the other cases with appreciable probability at *m* = 0 and 4.

The reduced variation in the methylation level among the receptor population would enhance chemotactic sensitivity. To test that, we performed simulations of the dose-response curves using the Ising-type model for receptor cooperativity (see supplemental information for details of the simulation).^65^ We simulated the four cases with an average methylation level of 2. The dose-response curves for the four cases are shown in Figure S8. We fit the curves with a Hill function, and the Hill coefficient $n_{H}$ was a good indicator of chemotactic sensitivity. $n_{H}$ for the four cases are 1.31 ± 0.12, 2.46 ± 0.29, 1.07 ± 0.11, and 4.83 ± 0.13 for cases 1 to 4, respectively, demonstrating the highest sensitivity for Case 4.

Therefore, encounter-rate matching of the sequential modifiable sites can minimize the probability of saturating the methylation sites, enhance chemotactic sensitivity, and also ensure robustness of methylation to noise in kinase activity and CheR/CheB expression.

## Discussion

We studied the chemotactic adaptation of the Tar-only strain using the CheZ-eCFP and CheY-eYFP FRET assay. We found a strong dependence of the methylation rate on the methylation level for the Tar-only strain and explained it based on the mechanism of TPM for the CheR molecules. To further validate this mechanism, we measured the adaptation of the Tar-only strains with different tethering chain lengths in Tar. The results of our measurements were consistent with previous Brownian dynamic simulation of tethered CheR motion,^56^ and consistent with the size of the CheR molecule or the distance between the catalytic and pentapeptide-binding sites on CheR measured by electron microscopy.^61^

The essence of the mechanism of the strong dependence due to TPM of CheR is as follows. Due to the volume-exclusion effects, the positions of CheR follow a translated Gaussian distribution, with the peak position of the distribution (the distance from the tethering point *R* = 10.5 nm) larger than the most distant methylation site #3 (*R* = 6.75 nm). Therefore, the dependence of the methylation rate on the methylation level follows the tail of a Gaussian distribution, resulting in imprecise adaptation in the Tar-only strain. Zero-activity adaptation experiments under saturated stimulation directly confirmed this dramatic relationship between methylation rate and methylation level directly.

Our measurements of the Tar-only strains with different tethering chain lengths in Tar demonstrated a linear relation between peak position $x_{0}$ of the translated Gaussian distribution of CheR concentration and the length of the flexible chain. The linker length varies among chemoreceptors. Specifically, the Tar receptor possessed a 34-aa flexible chain, while the Tsr receptor possessed a 30-aa flexible chain (counting I517 as the beginning of the tether). The linker length is intrinsically related to the location of methylation sites. In *E. coli*, the distances of Tar methylation sites from the tethering site (R514) were 4.65 nm, 5.7 nm, 6.75 nm and 3.45 nm, with an average distance of 5.14 nm. In contrast, the distances of Tsr methylation sites from the tethering site (R516) were 4.95 nm, 6.0 nm, 7.05 nm, 3.45 nm and 2.1 nm, with an average distance of 4.71 nm. Therefore, the average distance between the methylation sites and the tethering site in Tsr is reduced compared to that in Tar; consistently, the linker length in Tsr is also reduced.

The phenomenon of imprecise adaptation has been observed previously at high concentrations of serine (a ligand sensed by Tsr) and high concentrations of aspartate or its non-metabolizable analogue MeAsp, which are sensed by Tar. This imprecision has been attributed to the saturation of available modification sites on the receptors.^40^^,^^41^^,^^42^^,^^43^^,^^44^ Here, we observed that the imprecise adaptation for the Tar-only strain is manifested to a similar extent over a wide range of MeAsp concentrations. However, the model proposing saturation of available modification sites could not fit our data well. Instead, we explained the imprecision using the TPM model of CheR. In the wild-type strain with mixed types of receptors, the other types of receptors in the assistance neighbor supplied ample methylation sites to avoid a drastic change in the average methylation level of Tar receptors during the step response. As a result, the net average methylation rate does not change much, thereby alleviating the imprecision (see supplemental information and Figure S11 for a detailed simulation comparing the Tar-only and wild-type strains).

Previous experiments established sequentiality among the different methylation sites on the receptor.^35^^,^^36^^,^^37^^,^^38^^,^^39^ It was suggested that the methylation process for methylation sites 3, 2, and 1 of Tar is mostly sequential, and that the molecular mechanism of this sequential modification may be derived from the specific modification process at the modifiable sites of chemotactic receptors, in which methylation of the current site is affected by a residue 7 amino acids to the C-terminus. While both our work and ref. 39 study the adaptation of chemoreceptors, our focus differs. Ref. 39 focused on the effect of the sequential modification on adaptation precision and response gain, whereas we focused on the dynamic process of methylation and explored the effect of the diffusive motion of tethered CheR on adaptation. We employed the previously discovered sequentiality, and our results also offered an additional mechanism for maintaining the sequentiality. Furthermore, in our extraction of the methylation level from the receptor activity, we used the MWC model (Equation 1) with a linear form of the methylation-dependent free energy *f*(*m*). A previous study suggested that changes in methylation at individual sites of a receptor have different effects on receptor activity.^66^ However, the sequentiality of the methylation sites was well established, and the linear form of *f*(*m*) was measured experimentally;^67^ thus, the main effect of the differential effects of the methylation sites on receptor activity was automatically included in our extraction by using the experimentally measured *f*(*m*).

The mechanism of TPM for adaptation we discovered here resulted in a near-exponential distribution of CheR concentration along the sequential modifiable sites, so that CheR molecules encounter the preceding sites much more frequently than the ensuing sites. Through analysis of a sequential multisite catalytic reaction model, we found that this encounter-rate matching of the sequential modifiable sites results in the minimal cooperativity of *P*_*m=4*_ with respect to *K*_*eq*_*[R*_*tot*_*]/[B*_*tot*_*]* with a Hill coefficient near 1. Interestingly, this is in contrast to a previous multisite phosphorylation model in cell signaling,^62^ where higher cooperativity is needed and thus accomplished using a higher value of phosphorylation rates for larger *i*. In bacterial chemotaxis adaptation here, low cooperativity is needed and accomplished using a lower value of *k*_*i*_*[R*_*i*_*]* for larger *i*. This low cooperativity ensures robustness of methylation to fluctuations in kinase activity and cell-to-cell variations in CheR/CheB expression.

Moreover, we found that this encounter-rate matching results in a sharply peaked distribution of methylation levels at each *<m>*, thereby reducing variation of methylation levels among the receptor population. This minimizes catalytic deficiency at the methylation boundaries (*m* = 0 and 4) (by minimizing futile methylation at *m* = 4 and demethylation at *m* = 0),^65^ and also enhances chemotactic sensitivity.

### Limitations of the study

This work was based on measurements of receptor kinase activity of cell populations, using a general chemotaxis model to calculate the relationship between the methylation level and the methylation rate. The TPM model of CheR was subsequently established using the biochemical structure of the receptor. Future research may yield techniques for directly measuring the methylation kinetics of individual methylation sites, and more detailed quantitative results will further refine our model. Another limitation of this study is that we have not studied the effect of tethered CheB motion on bacterial adaptation.

## STAR★Methods

### Key resources table

**Table:** 

REAGENT or RESOURCE	SOURCE	IDENTIFIER
**Bacterial and virus strains**		

HCB1288	Howard Berg Lab	N/A
HCB1414	Howard Berg Lab	N/A

**Chemicals, peptides, and recombinant proteins**		

tryptone	Oxoid	CAT# LP0042B
IPTG	Sigma-Aldrich	CAT# I6758
Lactic acid	Sigma-Aldrich	CAT# 252476
Sodium chloride	Sigma-Aldrich	CAT# S9888

**Software and algorithms**		

Custom script	This study	https://github.com/CZhang2023/FRET_ana
Matlab R2020b	MathWorks	https://www.mathworks.com/products/MATLAB.html

### Resource availability

#### Lead contact

Further information and requests for resources and reagents should be directed to and will be fulfilled by the lead contact, Junhua Yuan (jhyuan@ustc.edu.cn).

#### Materials availability

This study did not generate new unique reagents.

### Experimental model and subject details

#### Strain and plasmids

HCB1288 [Δ*cheY cheZ*] and HCB1414 [Δ*tar tap tsr trg aer cheY cheZ*] are derivatives of *E. coli* K12 strain RP437. The plasmid pVS88 expresses CheY-eYFP and CheZ-eCFP under an IPTG-inducible promoter. The plasmid pLC113 and pPA114 express wild-type Tar and Tsr under a salicylate-inducible promoter respectively. The wild-type Tar receptor contains a flexible chain of 34 amino acids. As shown in Table 1, we deleted codons corresponding to the 544^th^ to 548^th^, and 537^th^ to 548^th^ amino acids from wild-type *tar*, and cloned them into a pBAD33 vector under an arabinose-inducible promoter, yielding pCZ1 and pCZ2, respectively. We inserted the sequence corresponding to the 12 amino acids from upstream of the pentapeptide of Tsr into the corresponding position of *tar*, and cloned it into a pBAD33 vector under an arabinose-inducible promoter, yielding pCZ4. HCB1288 transformed with pVS88, HCB1414 transformed with pVS88 and pLC113, and HCB1414 transformed with pVS88 and pCZ1, pCZ2, or pCZ4 were used to perform FRET experiments. Cells were grown in TB (1% (w/v) tryptone, 0.5% (w/v) NaCl, supplemented with the appropriate inducers and antibiotics: 0.1 mM IPTG, 1 μM salicylate, 100 μg/ml ampicillin, 25 μg/ml chloramphenicol) at 33°C to an optical density at 600 nm wavelength (OD_600 nm_) of about 0.45, and were then washed and suspended in motility medium (10 mM potassium phosphate, 0.1 mM ethylenediaminetetraacetic acid (EDTA), 10 mM lactic acid, and 1 μM methionine at pH 7.0).

### Method details

#### FRET assay and data analysis

We followed the receptor kinase activity *in vivo* by monitoring the FRET signal between the response regulator CheY and its phosphatase CheZ, which were fused with eYFP and eCFP, respectively, using a FRET setup described previously.^68^ The dose-response and step-response experiments were performed with a flow rate above 300 μl/min. We could switch the flowing solution between MeAsp and motility medium via a valve. When we changed the flow solution from MeAsp to motility medium, the stimulus was removed. The dose-response curves we measured for the wild-type and Tar-only strains are shown in Figure S9A. The pre-stimulus activity was calculated from the responses to stepwise addition and removal of a saturated stimulus. We fit our data with the MWC model of receptor cooperativity^47^ to extract the size of the receptor cluster *N* as described in Equation 1. We used $\alpha =-1.875,m_{0}=1.0,K_{off}=0.0182\;\text{mM}\;\text{and}\;K_{on}=3\;\text{mM}$ (for MeAsp) from previous works.^21^^,^^67^ According to the dose-response curves, the sizes of receptor cluster *N* were extracted to be 5.9±0.4 and 8.7±0.5 for the wild-type and Tar-only strains, respectively.

Our data were analyzed with a custom script in MATLAB (https://github.com/CZhang2023/FRET_ana). We determined the threshold concentration of saturating MeAsp using the dose-response curve of each strain. Then we picked a saturating concentration above the threshold (> 30 μM), and converted the FRET values to kinase activity *a* by measuring the full range of ΔFRET when adding, allowing for full adaptation, and then removing the saturating concentration of MeAsp, which corresponded to the receptor-kinase activity changing from 0 to 1. The pre-stimulus activity $a_{0}$ was then determined. In measuring the step responses, the FRET values were converted to kinase activity *a* by using the pre-stimulus FRET value and the lowest FRET value after the stepwise addition of a saturated concentration of MeAsp, which corresponded to activities of $a_{0}$ and 0, respectively. The relations between the adapted activity and MeAsp concentration for the wild-type and Tar-only strains are shown in Figure S9B.

To accurately obtain the highest activity *a*_*r*_ after removing the stimulus in the zero-activity adaptation assays, experiments were performed with a flow rate of 800 μl/min. To determine the upper limit of the waiting time *Δt*, we measured the mean duration of zero activity (before activity recovered above zero) after stepwise addition of saturating attractant, resulting in 139±25 s and 284±27 s for the wild-type and Tar-only strains under stepwise addition of 250 μM MeAsp, respectively. Accordingly, in measuring the methylation rates we used Δ*t* < 114 s and Δ*t* < 257 s for the wild-type and Tar-only strains, respectively, and this also ensured that $a_{r}<0.9$ for both strains. For each experiment with a specific value of Δ*t*, the peak value after removal of the saturated stimulus was identified and transformed to the methylation level of receptors with Equation 1. The average methylation rate was calculated as the change in methylation level during the zero-activity state divided by methylation time Δ*t*.

## Data Availability

The authors declare that all data supporting the findings of this study are available in the article along with supplemental information data. The custom analysis script in MATLAB is available at: https://github.com/CZhang2023/FRET_ana.
