# Supplementary material for: Tethered particle motion of the adaptation enzyme CheR in bacterial chemotaxis
Source: iScience. 2023 Sep 17;26(10):107950. doi: 10.1016/j.isci.2023.107950 (PMC10561060; doi:10.1016/j.isci.2023.107950)
Supplement: Document S1. Figures S1–S11 [file mmc1.pdf]

iScience, Volume *26*

Supplemental information

Tethered particle motion of the adaptation enzyme CheR in bacterial chemotaxis

Caijuan Yue, Chi Zhang, Rongjing Zhang, and Junhua Yuan

Supplemental Text

The analytical model of multisite catalytic reaction using the local adaptation (LA) scheme [S1].

In the LA scheme, the methylation/demethylation rate depends on the activity of the individual receptor itself a_m $(m = 0,1,2,3,4)$ instead of the average activity of the group $\langle a \rangle$. We write out the dependence explicitly, and the rate equations are:

$$\frac{d[X_0]}{dt} = k_{-1}a_1[B_1][X_1] - k_1(1 - a_0)[R_1][X_0],$$

$$\frac{d[X_i]}{dt} = k_{-(i+1)}a_{i+1}[B_{i+1}][X_{i+1}] - k_{i+1}(1 - a_i)[R_{i+1}][X_i] + k_i(1 - a_{i-1})[R_i][X_{i-1}] - k_{-i}a_i[B_i][X_i],$$

where i = 1 to 3

$$\frac{d[X_4]}{dt} = k_4(1 - a_3)[R_4][X_3] - k_{-4}a_4[B_4][X_4],$$

where $[X_i]$ is the concentration of receptors with a methylation level of i, and a_i is the activity of the receptors with a methylation level of i. k_i and k_{-i} represent the reaction rate constants of methylation and demethylation for site i, respectively. $[R_i]$ and $[B_i]$ denote the concentrations of CheR and CheB-P at site i, respectively. At steady state when the adaptation was complete, the rate of change for the concentration of each methylated form was zero. Thus,

$$k_i(1 - a_{i-1})[R_i][X_{i-1}] = k_{-i}a_i[B_i][X_i], \quad \text{(S1)}$$

where $i = 1, 2, 3, 4$. We can then calculate the probability of receptors with a methylation level of i ($P_{m=i}$):

$$P_{m=i} = \frac{T_i}{\sum_{i=0}^{4} T_i}, \quad \text{(S2)}$$

with

$$T_i = \begin{cases} 1 & i = 0 \\ \prod_1^i \frac{\theta P_i(1 - a_{i-1})}{a_i} & i > 1 \end{cases}$$

where $\theta = \frac{k_i[R_{tot}]}{k_{-i}[B_{tot}]}$ is independent of i. $[R_i] = [R_{tot}] * P_i$ and $[B_i] = [B_{tot}]$. $[B_{tot}]$ and $[R_{tot}]$ represent the concentrations of CheB-P and CheR in a cell, respectively. P_i denotes the proportion of $[R_{tot}]$ at the ith site, and $\sum_{i=1}^{4} P_i = 1$. a_i can be calculated by

$$a_{m=0,1,2,3,4} = \frac{1}{1 + \exp(E^M + E^C)}, \quad (S3)$$
$$E^M = \alpha(m - 1.0),$$
$$E^C = C_J(\langle a \rangle - 0.5),$$

where E^M and E^C represent the methylation-dependent and receptor coupling-dependent free energy for the receptor with methylation level $m = 0,1,2,3,4$, respectively. The average activity of all receptors $\langle a \rangle = \sum_{i=0}^{4} P_{m=i} a_{m=i}$. Then, we used the *fsolve* function of MATLAB R2018b to solve the nonlinear system of ten equations composed of Eqs. S2 and S3 for each value of θ. We used $\alpha = -1.875$, $C_J = -2.5$ here. The results for four cases of probability (P_i) sets described in the main text are shown in Fig. S10. Case 4 for encounter-rate matching of the sequential modifiable sites exhibits the lowest and a nearly constant standard deviation for $0 < \langle m \rangle < 4$ among all cases, and exhibits the highest threshold $\langle m \rangle$ for saturating the methylation sites. This holds for other values of C_J.

Therefore, the conclusions that encounter-rate matching of the sequential modifiable sites can minimize the probability of saturating the methylation sites and ensure robustness of methylation to noise in kinase activity and CheR/CheB expression still stand in the LA scheme.

Simulation of dose-response curve for the four cases of CheR encounter probability settings

To investigate the effect of different distributions of methylation level on the sensitivity of receptor activity response, we simulated the dose-response relationship for the four distributions of the methylation level in Fig. 5I-L that corresponded to cases 1 to 4. Based on the Ising-model for receptor cooperativity [S2], we considered the average activity change of a 100×100 Tar receptor array in response to different concentrations of MeAsp. Each receptor in the array could be either active (*a*=1) or inactive (*a*=0), with the probability determined by the free energy f:

$$f = E_i^L + E_i^M + E_i^C,$$

where E_i^L, E_i^M and E_i^C represent the ligand-dependent, methylation-dependent and receptor coupling-dependent free energies for the *i*th receptor, respectively. They can be calculated by

$$E_i^L = \ln\frac{1 + L/K_{off}}{1 + L/K_{on}}$$

$$E_i^M = \alpha(m - 1.0)$$

$$E_i^C = \sum_j C_J(a_j - 0.5),$$

where L denotes the ligand concentration, m=0,1,2,3,4 denotes the methylation level, and K_{off} and K_{on} are the dissociation constants for the inactive and active receptors, respectively. The activity of the *i*th receptor can be affected by the activities of its neighbors by introducing a coupling constant C_J. a_j represents the activity of the *j*th neighbor, and the neighbors of the *i*th receptor include four receptors located up, down, left and right to it in the receptor array.

The probability of the *i*th receptor in the activated state is

$$P = \frac{1}{1 + \exp(f)}.$$

In our simulation, we used the parameters $K_{off} = 0.0182\,mM$, $K_{on} = 3000\,mM$, $\alpha = -1.875$, $C_j = -2.5$. We also set $E_i^M = 3.75$ for $m = 0$ to further suppress the activity of the receptor at this state, as was done previously [S1]. The average methylation level was kept at $\langle m \rangle = 2$, and the distributions of methylation levels for individuals were generated from Fig. I-L for cases 1 to 4. The results are shown in Fig. S4.

Simulation of perfect adaptation for the wild-type strain

In light of the imprecise adaptation due to the tethered particle motion of CheR for the Tar-only strain, we sought to explain the near perfect adaptation for the wild-type strain with mixed receptors. We performed simulations combining a stochastic process of methylation/demethylation and the assistance neighborhoods (ANs) model for adaptation [S3]. The wild-type receptor-cluster was modelled as a MWC cluster with

18 receptors, composed of 6 Tar and 12 Tsr receptors that was consistent with the experimentally measured ratio for the wild-type strain [S4]. Each receptor has four modifiable sites. The activity of the MWC cluster can be described by

$$a_{cluster} = \frac{1}{1 + exp(\sum_1^N (f_L + f_m))},$$

where the summation was over all of the N=18 receptors. We defined three assistance neighborhoods of six receptors in line with the experimental measurement [S5]. The free energy of each receptor is the sum of f_L and f_m that can be calculated with equations in the main text. The dissociation constants of Tsr for MeAsp are $K_s^{on} = 100\ mM$ and $K_s^{off} = 10^6\ mM$ for the on and off states, respectively. For the adaptation process, the Gillespie algorithm was used to determine the time of the next methylation/demethylation [S6]. In each modification, receptors in each neighborhood were equally likely to be selected. They would be methylated or demethylated with a rate of $g_R \times (1 - a_{cluster})$ or $g_B \times a_{cluster}$, respectively. We use $g_R = 0.1\ s^{-1}$ and $g_B = 2 \times g_R$ to set an adapted activity of about 1/3. To reproduce the imprecise adaptation in the Tar-only strain, we used the measured probability of methylation for the four sites (the translated Gaussian distribution), with an equal probability of demethylation for each site. After each reaction, the methylation state of the receptors was updated, and the activity of the cluster was changed accordingly.

The simulation results are shown in Fig. S11. Each data point was the average of 200 runs of simulations, with each simulation followed for 2000 s. The Tar-only strain demonstrated an obvious imprecise adaptation, whereas the wild-type strain exhibited a near perfect adaptation.

The dependence of the methylation rate on the methylation level could be extended to Trg

The low-abundance chemoreceptors Trg and Tap lack the pentapeptide. Thus, the effective adaptation of Trg mainly results from the tethered CheR to Tsr or Tar in the

same trimer. Based on the structure of the trimer of homodimers, the cytoplasmic part of a trimer could be described as three rods connected at their cytoplasmic tips with an angle between rods of about 20° [S7]. As shown in Fig. S8A, the gray, orange and green rods represent the cytoplasmic domains of Trg, Tar and Tsr dimers that do not include the HAMP domains, respectively. The white circles on Trg denote the five modifiable sites. The blue curves are tethers of Tar or Tsr, and orange balls represent the adaptation enzymes (CheR). We investigated whether the modification of the five sites on Trg by the tethered CheR to Tar (or Tsr) will induce dependence of the methylation rate on the methylation level, that is, whether the tethered CheR molecules encounter the sites at quite different rates, by calculating the relative encounter rates of CheR (tethered to Tar) for the sites on Trg. As shown in Fig. S8B, five modifiable sites (E510, E501, E305, Q312 and Q319) are located on the Trg receptor [S8,9]. The length of the receptor (*OA* and *O'A*) is about 20 nm, and the angle between dimers ($\angle A$) is about 20°. Thus, the distance between the tethering points (*OO'*) is about 6.95 nm. The distances between each site and the tethering point of Trg (*O*) could be calculated as we did for the Tar receptors in the main text, which are 2.1 nm, 3.45 nm, 4.65 nm, 5.7 nm and 6.75 nm. Now we can calculate the distances between each site and the tethering point of Tar (*O'*).

$$O'P = \sqrt{(OP - OO' \cos 80°)^2 + (OO' \sin 80°)^2},$$

where *P* denotes any modifiable site of Trg. The concentration distribution of CheR as a function of the distance from the tethering point of Tar is

$$C_{CheR}(R) \propto \exp\left(\frac{-3(R - 10.5)^2}{2 \times 12.24 \times 0.76}\right).$$

Now we can substitute R with $O'P$. Thus, the relation between the relative concentration of CheR ($[\text{CheR}]_{\text{relative}}$, relative to $m = 1$) and methylation level is obtained, which is plotted in Fig. S6C. Therefore, the dependence of the methylation rate (proportional to the concentration of CheR) on the methylation level could also be applied to chemoreceptors lacking a pentapeptide.

Supplemental references:

1. Lan, G., Schulmeister, S., Sourjik, V., and Tu, Y. (2011). Adapt locally and act globally: strategy to maintain high chemoreceptor sensitivity in complex environments. Mol Syst Biol *7*, 475. https://doi.org/10.1038/msb.2011.8.
2. Duke, T.A.J., and Bray, D. (1999). Heightened sensitivity of a lattice of membrane receptors. Proc. Natl. Acad. Sci. USA *96*, 10104-10108. https://doi.org/10.1073/pnas.96.18.10104.
3. Endres, R.G., and Wingreen, N.S. (2006). Precise adaptation in bacterial chemotaxis through "assistance neighborhoods". Proc. Natl. Acad. Sci. USA *103*, 13040-13044. https://doi.org/10.1073/pnas.0603101103.
4. Yang, Y.L., and Sourjik, V. (2012). Opposite responses by different chemoreceptors set a tunable preference point in *Escherichia coli* pH taxis. Mol Microbiol *86*, 1482-1489. https://doi.org/10.1111/mmi.12070.
5. Li, M., and Hazelbauer, G.L. (2005). Adaptational assistance in clusters of bacterial chemoreceptors. Mol. Microbiol. *56*, 1617-1626. https://doi.org/10.1111/j.1365-2958.2005.04641.x.
6. Gillespie, D.T. (1995). The mathematics of Brownian motion and Johnson noise. https://doi.org/10.1119/1.18210.
7. Vaknin, A., and Berg, H.C. (2006). Osmotic stress mechanically perturbs chemoreceptors in *Escherichia coli*. Proc. Natl. Acad. Sci. USA *103*, 592-596. https://doi.org/10.1073/pnas.0510047103.
8. Kehry, M.R., Engström, P., Dahlquist, F.W., and Hazelbauer, G.L. (1983). Multiple covalent modifications of Trg, a sensory transducer of *Escherichia coli*. J. Biol. Chem. *258*, 5050-5055. https://doi.org/10.1016/S0021-9258(18)32536-5.
9. Bollinger, J., Park, C., Harayama, S., and Hazelbauer, G.L. (1984). Structure of the Trg protein: Homologies with and differences from other sensory transducers of *Escherichia coli*. Proc. Natl. Acad. Sci. USA *81*, 3287-3291. https://doi.org/10.1073/pnas.81.11.3287.
10. Mesibov, R., and Adler, J. (1972). Chemotaxis toward amino acids in *Escherichia coli*. Journal of bacteriology *112*, 315-326. https://doi.org/10.1128/jb.112.1.315-326.1972.
11. Sourjik, V., and Berg, H.C. (2004). Functional interactions between receptors in bacterial chemotaxis. Nature *428*, 437-441. https://doi.org/10.1038/nature02406.
12. Mello, B.A., and Tu, Y. (2005). An allosteric model for heterogeneous receptor complexes: understanding bacterial chemotaxis responses to multiple stimuli. Proc. Natl. Acad. Sci. USA *102*, 17354-17359. https://doi.org/10.1073/pnas.0506961102.

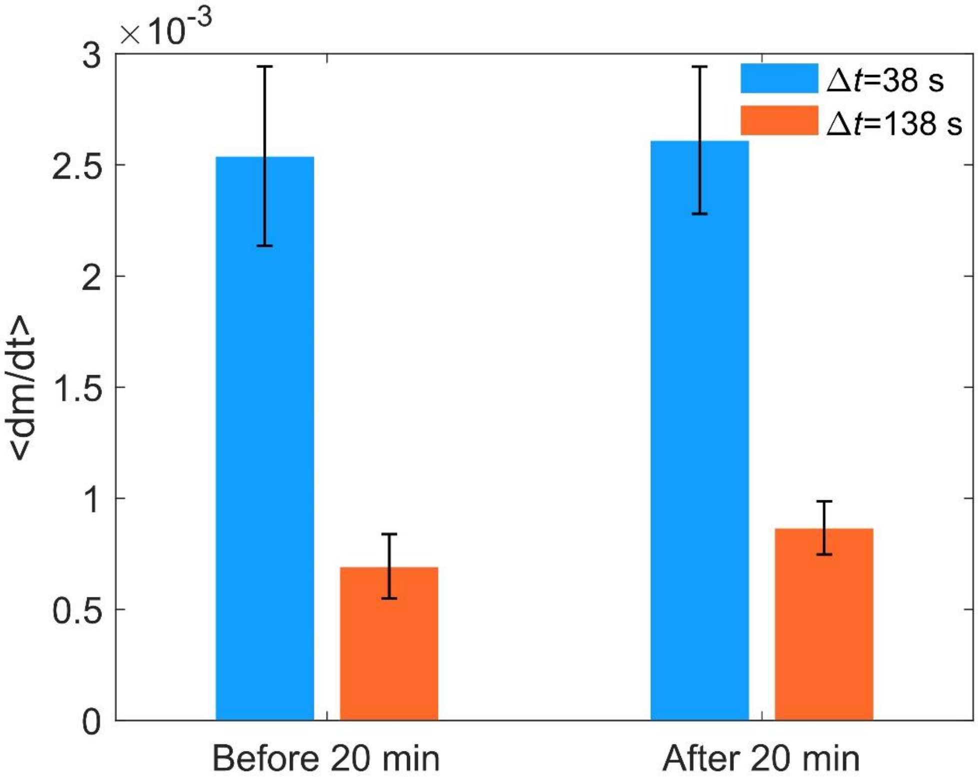

Figure S1. Comparison of the net average methylation rate $\langle dm/dt \rangle$ for two different values of Δt, both before and after a 20-min period, related to Figure 1. $\langle dm/dt \rangle$ for $\Delta t = 38$ s was 0.0025 ± 0.0004 and 0.0026 ± 0.0003 before and after 20 min, respectively, and $\langle dm/dt \rangle$ for $\Delta t = 138$ s was 0.0007 ± 0.0001 and 0.0009 ± 0.0001 before and after 20 min, respectively. The errors represent S. D.

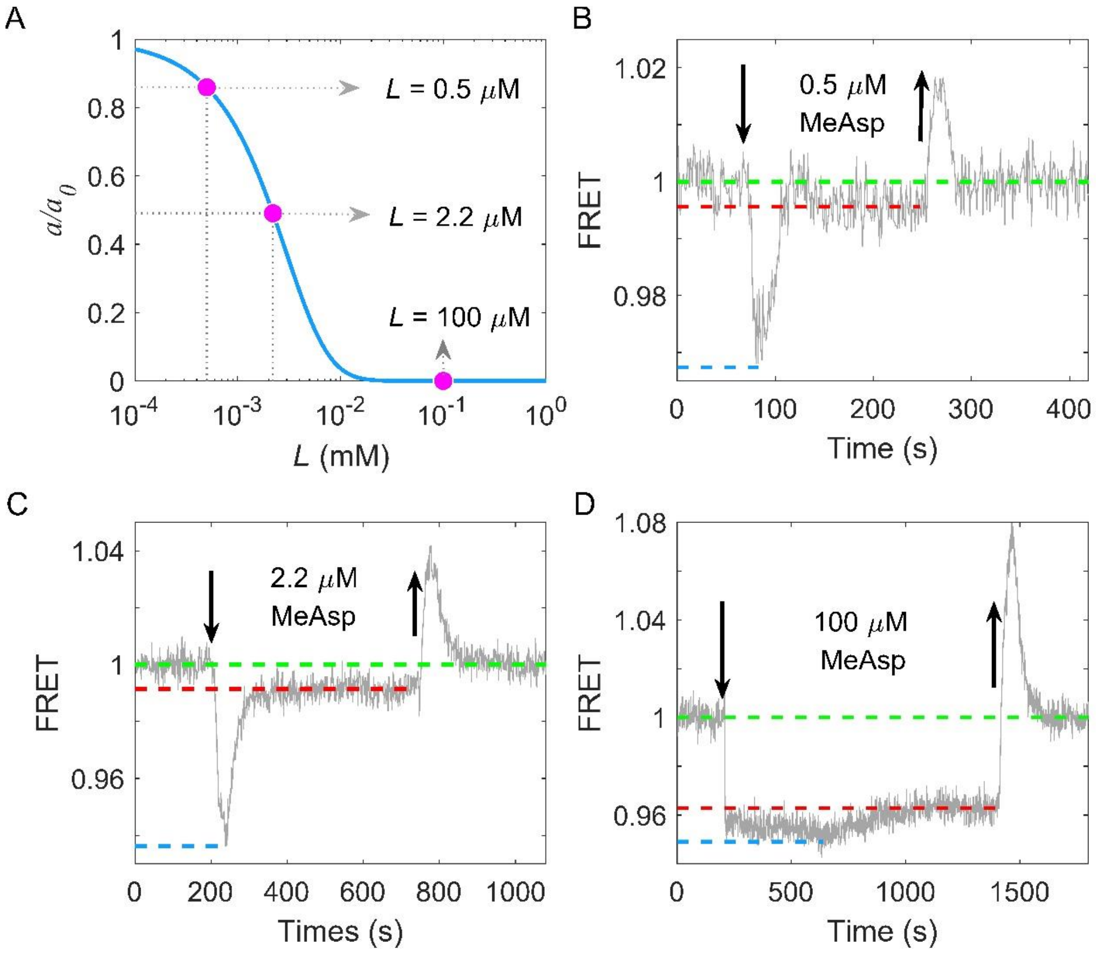

Figure S2. Three typical traces of step response to different concentrations of MeAsp for the Tar-only strain, related to Figure 1. The corresponding positions in the dose-response curve for the three MeAsp concentrations are shown in (**A**). (**B**)-(**D**) show the traces. a_0 denotes the pre-stimulus level of kinase activity. The FRET values represent the intensity ratio of YFP to CFP. The green dashed lines denote the pre-stimulus value. The red and blue dashed lines denote the adapted value and the response value, respectively. The addition and removal times of MeAsp are depicted with arrows.

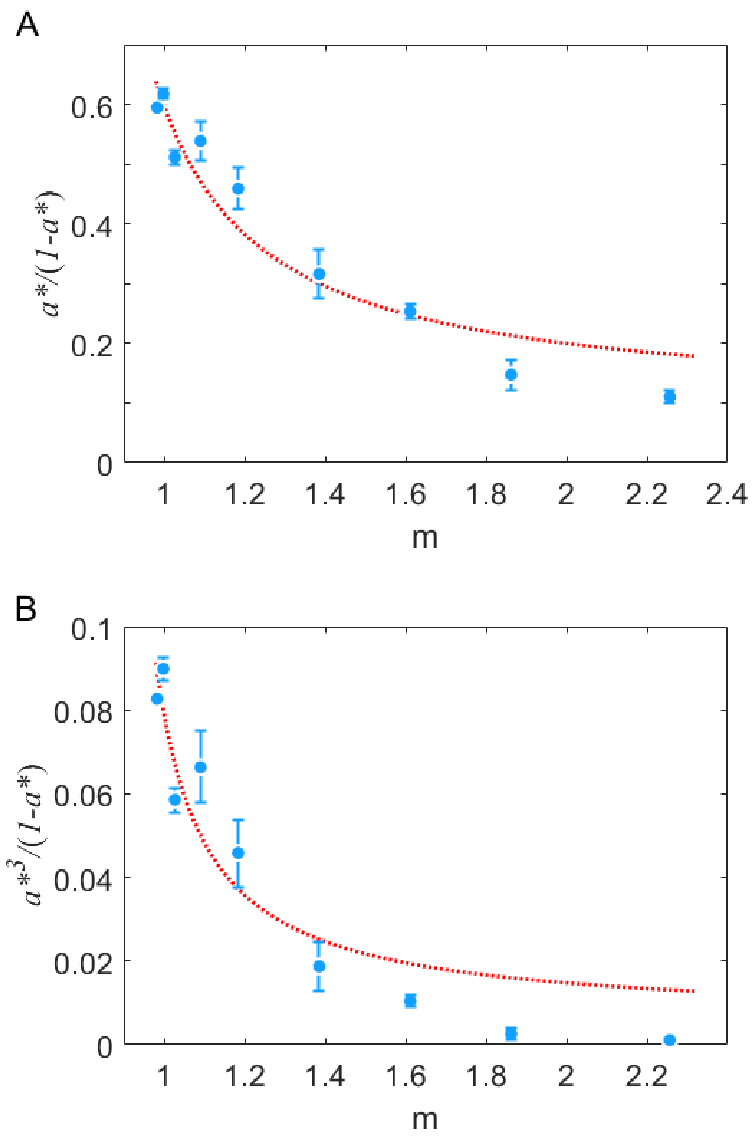

Figure S3. Fitting of the model of scarce methylation/demethylation sites to our data, using $\frac{dm}{dt} = g_R \frac{m_{max}-m}{m_{max}-m+K}(1-a^*) - g_B \frac{m}{m+K} a^* = 0$, related to Figure 2. (A) and $\frac{dm}{dt} = g_R \frac{m_{max}-m}{m_{max}-m+K}(1-a^*) - g_B \frac{m}{m+K} a^{*3} = 0$ **(B)**, where m_{max} is the maximum methylation level and K is used to implement reduced efficiency of methylation or demethylation due to the effect of scarce modifiable sites. The solid dots represent our experimental data with STD. The constraints of fitting (red dashed line) are $g_R/g_B > 0$, $m_{max} > 0$, $K > 0$. The fitting results are $m_{max} = 2.42*10^{-14}$ and $7.8*10^{-5}$, $K = 0.73$ and 0.86, $g_R/g_B = 0.09$ and 0.06 in (A) and (B), respectively. Even with unreasonable fitted values of m_{max}, the fits are not good.

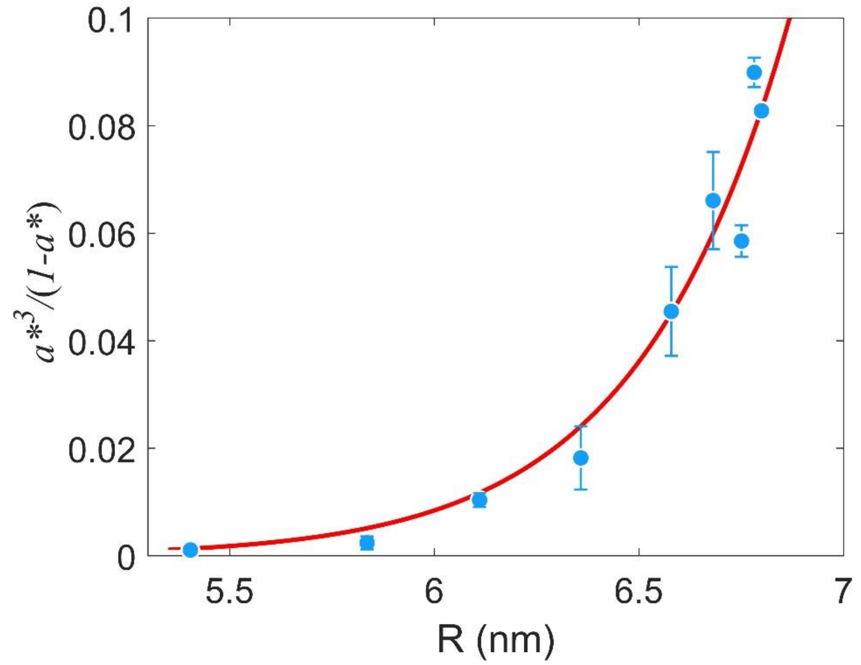

Figure S4. Fitting of the tethered-particle model based on the a^3 dependence of the demethylation rate, related to Figure 3. The blue dots are experimental data with the errors of SDs. The red solid line denotes the fit with $\frac{a^{*3}}{1-a^*} = c \times \exp\left(\frac{-3(R-x_0)^2}{2L_{total}b}\right)$ with $L_{total} = 12.24\ nm$ and $b = 0.76\ nm$.

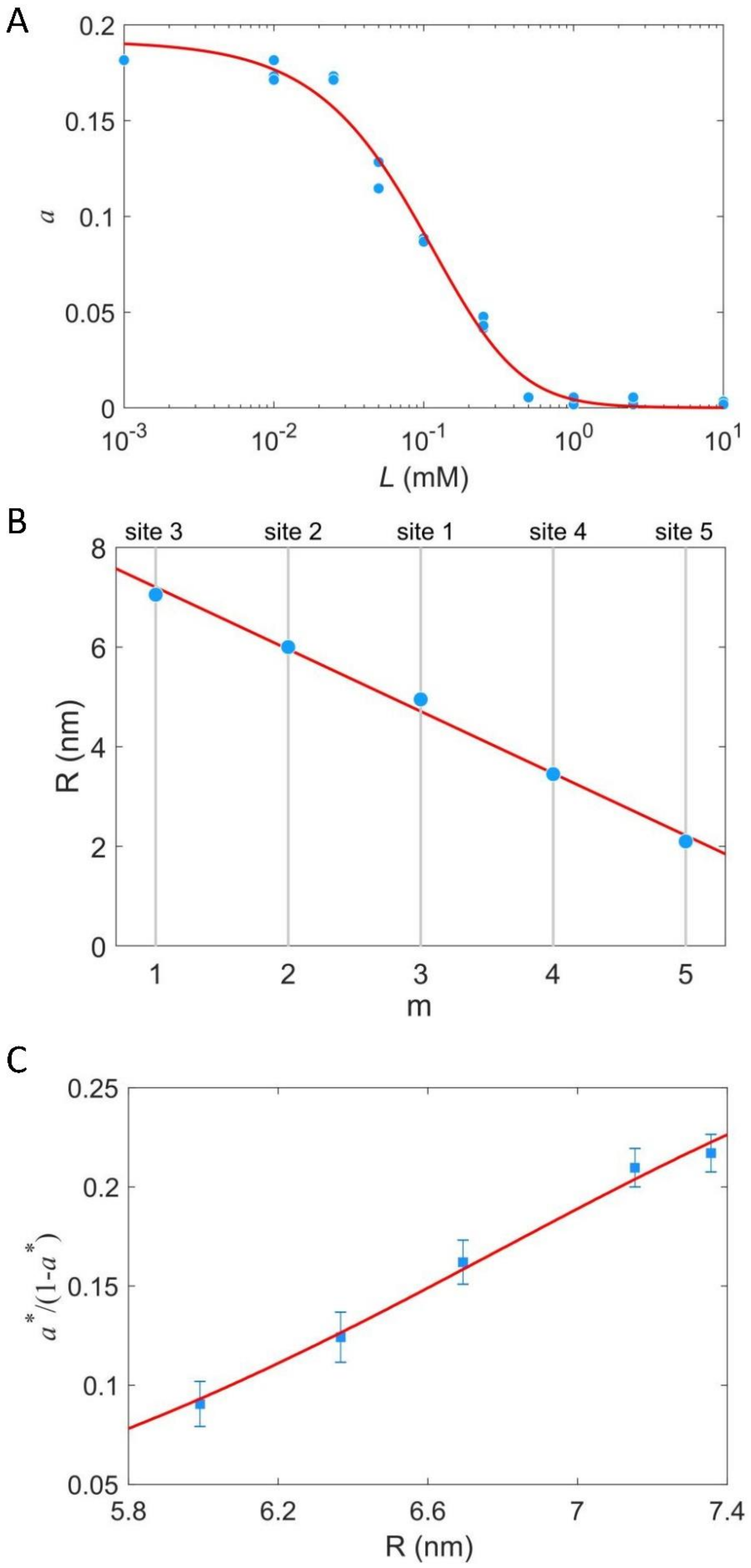

Figure S5. Application of the TPM model to measurements with the Tsr-only strain, related to Figure 3. **A.** The dose-response curve of the Tsr-only strain HCB1414-pPA114-pVS88 (1 µM salicylate) to 2-aminoisobutyric acid (a non-metabolic analogue

of serine [S10]). The solid lines are fits with the MWC model $a = \frac{1}{1+\exp\left(N\left(f_m+\ln\frac{1+L/K_{off}}{1+L/K_{on}}\right)\right)}$, where $\sqrt{K_{\mathrm{off}}K_{on}} = 1.26\ \mathrm{mM}$ is estimated from the activity change under 50% occupancy of Tsr, which is derived from the dose-response curve of the strain expressing only Tsr at 0.7 times the native level[S11] and the dissociation constants of serine for active (109.9 μM) and inactive (34.5 μM) conformations of Tsr[S12]. **B.** The relationship between the distance (R) of each modifiable site (Q297 E304 Q311 E493 E502) from the tethering point (R516) and the methylation level (m). The gray lines denote the modifiable sites. The red line is the fit with a linear function $R = -1.25m + 8.45$. **C.** Fitting of the TPM model (Eq. 8 in the main text) to the experimental data measured with the Tsr-only strain. The parameters are set as $b = 0.76\ \mathrm{nm}$ and $L_{total} = 0.36 \times 30 = 10.8\ \mathrm{nm}$. The fitted value of x_0 is 8.5 ± 0.2 nm (mean ± SD). The blue squares are the experimental data. The red line is the fit. Errors are SD.

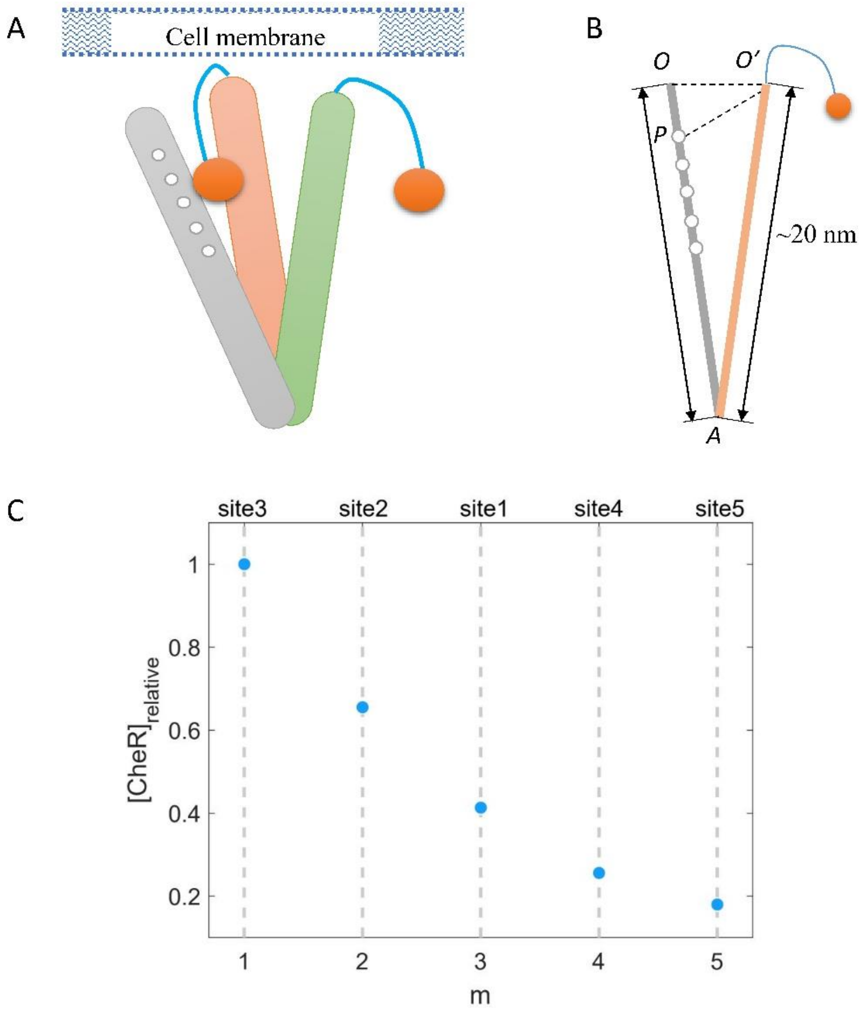

Figure S6. Dependence of the methylation rate on the methylation level for Trg, related to Figure 3. **A.** Schematic illustration of the cytoplasmic domain of a trimer that consists of Trg (gray rod), Tar (orange rod) and Tsr (green rod) dimers. The white circles on Trg denote the five modifiable sites. The blue curves are tethers of Tar or Tsr, and orange balls represent the adaptation enzymes (CheR). **B.** Geometric approximation of the two-dimensional spatial relation of the cytoplasmic domain of two receptors (Trg and Tar). **C.** The dependence of the relative concentration of CheR on the methylation level *m* for Trg.

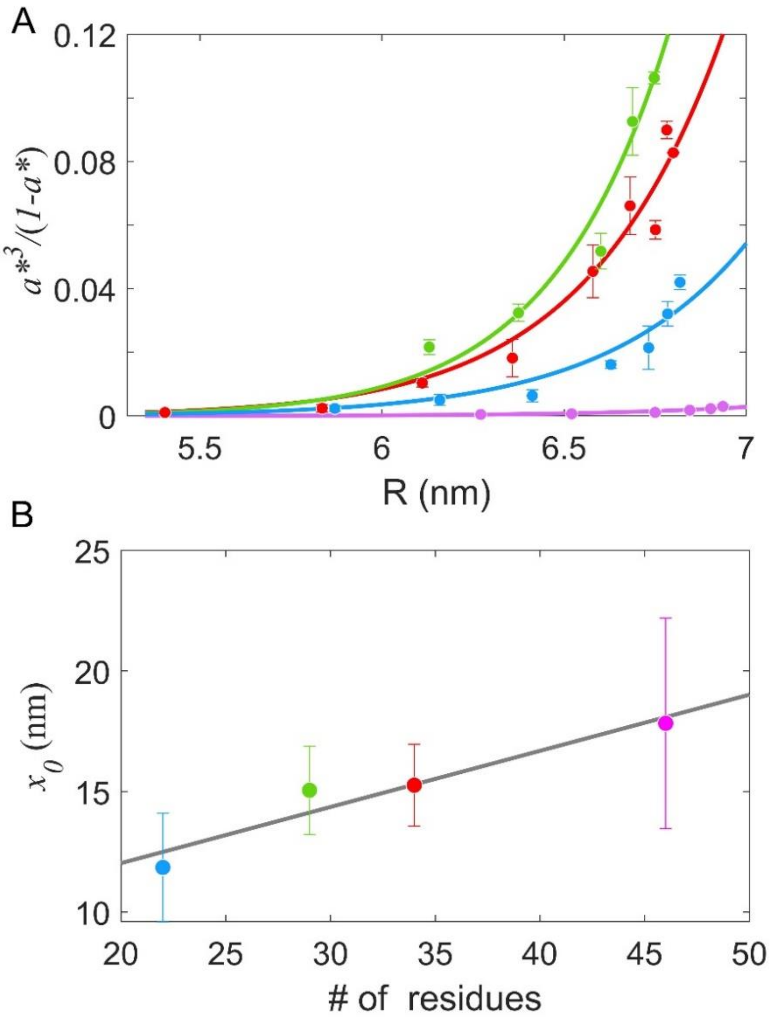

Figure S7. Fitting of the TPM model based on the a^3-dependence of the demethylation rate for Tar receptor with various lengths of the C-terminal flexible chain, related to Figure 4. **A.** Fitting of the tethered-particle model based on the a^3 dependence of the demethylation rate to experimental data with four Tar-only strains expressing different Tar constructs. Data with SDs from the strains with Tar receptors containing a flexible chain with 34, 29, 22 and 46 residues are represented by red, green, blue and purple dots, respectively. The fit result is represented by a solid line in the corresponding color. The fit function is $\frac{a^{*3}}{1-a^*} = c \times \exp\left(\frac{-3(R-x_0)^2}{2L_{total}b}\right)$ with $b =$ 0.76 nm, and $L_{total} =$ 12.24 nm, 10.44 nm, 7.92 nm and 16.56 nm for the chains with 34, 29, 22 and 46 residues, respectively. **B.** The relation between the fitted translational distance x_0 (extracted from **A** with SD) and the length of the flexible chain. The gray line is the fit with a linear function. The fitted slope and y intercept are 0.233 ± 0.100 nm/aa and 7.4 ± 3.4 nm, respectively.

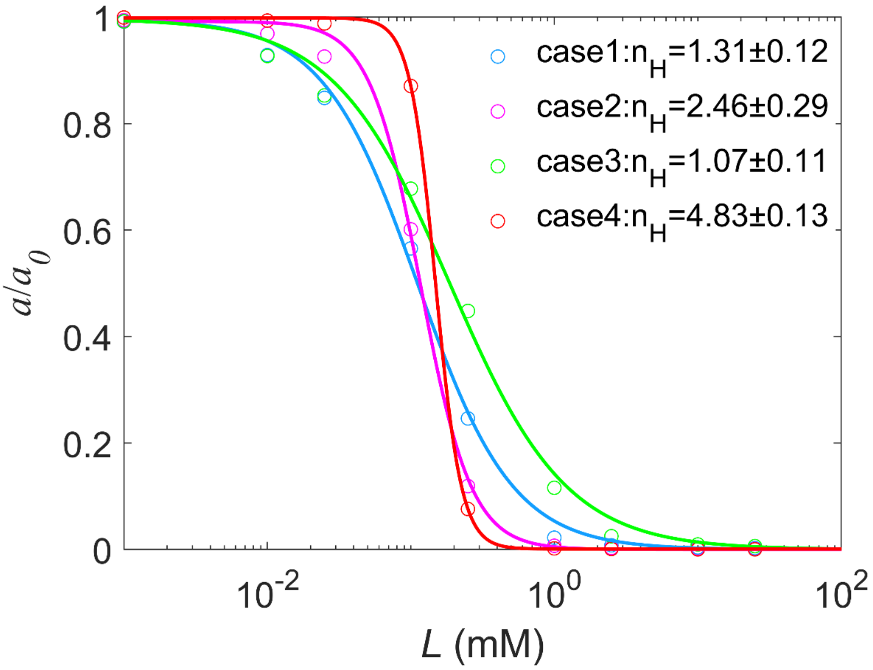

Figure S8. The simulated dose-response curves for the four cases of CheR encounter probability settings for the four sites with $\langle \boldsymbol{m} \rangle = 2$, related to Figure 5. The dots are the simulation results, and the solid lines are fits with a Hill equation. The Hill coefficients n_H are shown in the legend with SD. Different colors represent different cases.

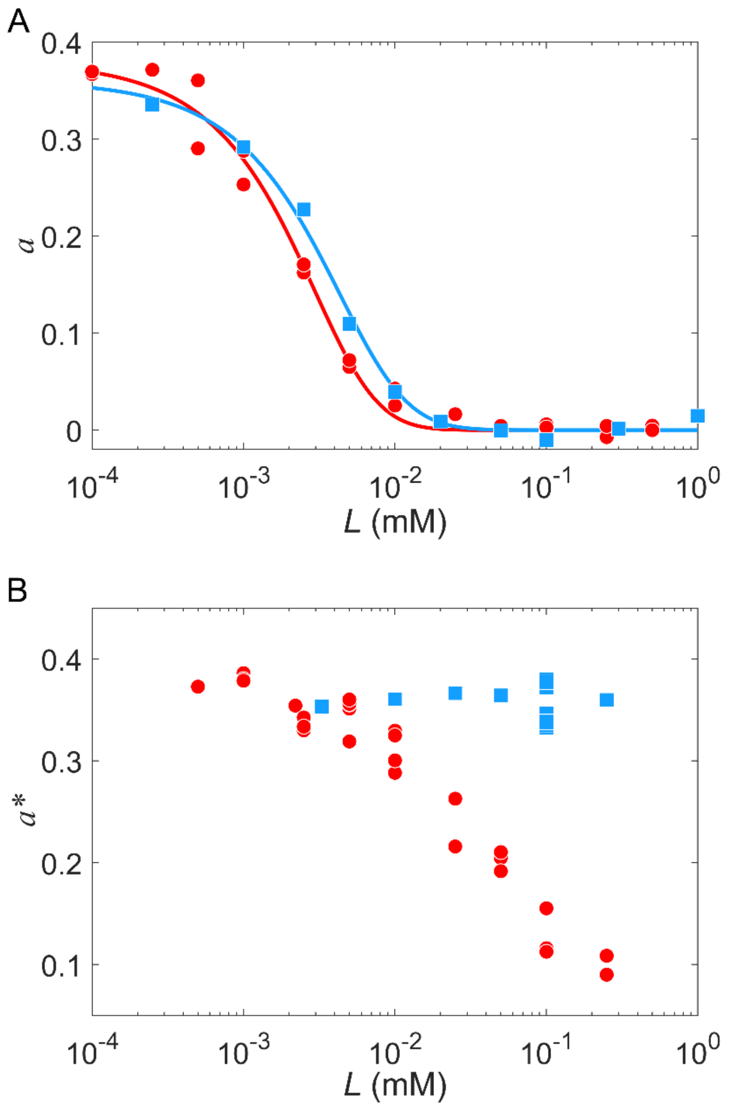

Figure S9. Comparison of the response and adaptation between the Tar-only strain and the wild-type strain, related to STAR Methods. **A.** The dose-response curves of wild-type strain (blue squares) and Tar-only strain (red dots). The solid lines are fits with the MWC model to extract the cluster size. **B.** The adapted activity of the wild-type strain (blue squares) and the Tar-only strain (red dots) after stepwise addition of different concentrations of MeAsp. Each data point represents the result of one step-response experiment.

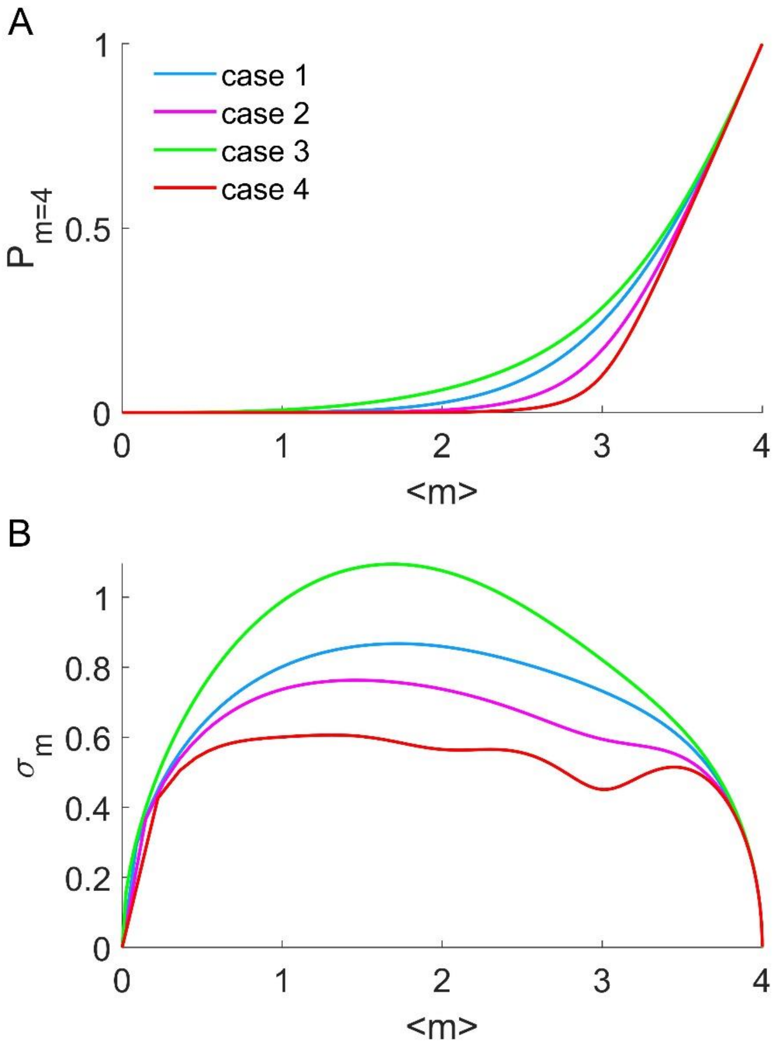

Figure S10. The analytical results of the multisite catalytic reaction based on the local adaptation (LA) scheme, related to Figure 5. **A.** The relation between the probability of saturated methylation sites and the average methylation level for the four cases. **B.** The standard deviation of the methylation level σ_m at different mean methylation levels $\langle m \rangle$. Case 1: $P_1=P_2=P_3=P_4=0.25$, blue line; Case 2: $P_1=7/16$, $P_2=5/16$, $P_3=3/16$, $P_4=1/16$, pink line; Case 3: $P_1=1/16$, $P_2=3/16$, $P_3=5/16$, $P_4=7/16$, green line; Case 4: $P_1=0.7809$, $P_2=0.1856$, $P_3=0.0309$, $P_4=0.0026$, red line.

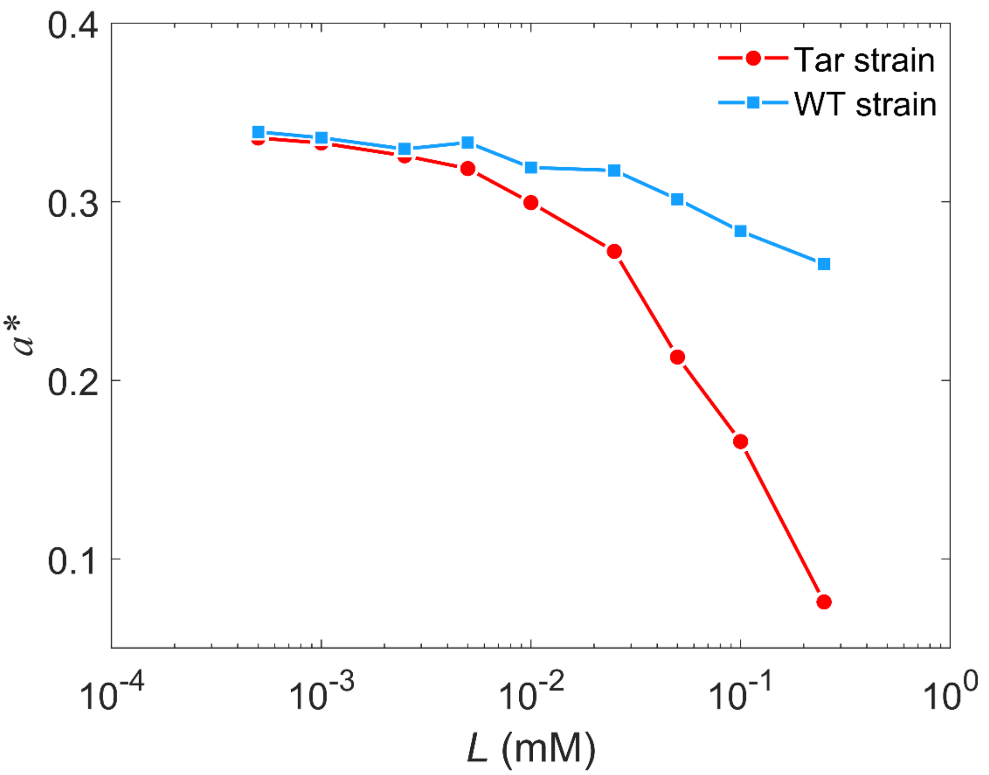

Figure S11. The simulated adapted activity of the wild-type (blue squares) and the Tar-only strain (red dots) at different concentrations of MeAsp, related to Discussion. We use an assistance neighborhood of 6 receptors. Each data point is the average value of 200 runs of simulation.
